# CircUGP2 Suppresses Intrahepatic Cholangiocarcinoma Progression via p53 Signaling Through Interacting With PURB to Regulate ADGRB1 Transcription and Sponging miR‐3191‐5p

**DOI:** 10.1002/advs.202402329

**Published:** 2024-08-09

**Authors:** Rui Xiang Chen, Shuo Chen Liu, Xue Chun Kan, Yi Rui Wang, Ji Fei Wang, Tian Lin Wang, Chang Li, Wang Jie Jiang, Yan An Lan Chen, Tao Zhou, Shi Long Fan, Jiang Chang, Xiao Xu, Kuang Heng Shi, Yao Dong Zhang, Ming Yu Wu, Yue Yu, Chang Xian Li, Xiang Cheng Li

**Affiliations:** ^1^ Hepatobiliary Center The First Affiliated Hospital of Nanjing Medical University Key Laboratory of Liver Transplantation Chinese Academy of Medical Sciences NHC Key Laboratory of Living Donor Liver Transplantation (Nanjing Medical University) Nanjing Jiangsu 210029 China; ^2^ School of Medicine Southeast University Nanjing Jiangsu 210009 China; ^3^ The Affiliated Wuxi People's Hospital of Nanjing Medical University Wuxi People's Hospital Wuxi Medical Center Nanjing Medical University Wuxi Jiangsu 214023 China

**Keywords:** ADGRB1, circUGP2, intrahepatic cholangiocarcinoma, lipid nanoparticles, p53 signaling, PURB

## Abstract

Intrahepatic cholangiocarcinoma (ICC) is the second most common primary liver cancer and its prognosis remains poor. Although growing numbers of studies have verified the involvement of circular RNAs (circRNAs) in various cancer types, their specific functions in ICC remain elusive. Herein, a circRNA, circUGP2 is identified by circRNA sequencing, which is downregulated in ICC tissues and correlated with patients’ prognosis. Moreover, circUGP2 overexpression suppresses tumor progression in vitro and in vivo. Mechanistically, circUGP2 functions as a transcriptional co‐activator of PURB over the expression of ADGRB1. It can also upregulate ADGRB1 expression by sponging miR‐3191‐5p. As a result, ADGRB1 prevents MDM2‐mediated p53 polyubiquitination and thereby activates p53 signaling to inhibit ICC progression. Based on these findings, circUGP2 plasmid is encapsulated into a lipid nanoparticle (LNP) system, which has successfully targeted tumor site and shows superior anti‐tumor effects. In summary, the present study has identified the role of circUGP2 as a tumor suppressor in ICC through regulating ADGRB1/p53 axis, and the application of LNP provides a promising translational strategy for ICC treatment.

## Graphical abstract

Intrahepatic cholangiocarcinoma is the second most prevalent primary liver cancer with a poor prognosis. This study identifies a novel mechanism underlying ICC progression by circUGP2‐mediated ADGRB1/p53 axis activation. This effect is achieved by circUGP2 interacting with PURB to co‐activate ADGRB1 transcription, and sponging miR‐3191‐5p to upregulate ADGRB1 expression. The LNP system loading circUGP2 plasmids provides a promising strategy for ICC treatment.

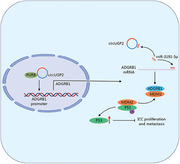

## 1 Introduction

Intrahepatic cholangiocarcinoma (ICC), accounting for 20% of all hepatic malignancies, is the second most common primary liver cancer with a rising incidence over recent decades.^[^ ^1^ ^]^ The majority of ICC patients present with advanced‐stage, rendering surgical resection unfeasible.^[^ ^2^ ^]^ Even among those undergoing curative resection, the recurrence rate remains high, with the 5‐year overall survival rate estimated at ≈25%–40%.^[^ ^3^ ^]^ Although recent advancements, including systemic chemotherapy, targeted therapy, and immunotherapy, have led to improvements in patients’ outcomes, survival remains poor.^[^ ^4^ ^]^ Therefore, there is an urgent need to delve into the molecular mechanisms of ICC and develop new treatment strategies to improve patients’ survival.

As is well known, the p53 protein is a transcription factor and functions as a tumor suppressor by targeting multiple genes involved in the regulation of various cellular processes, such as cell cycle, apoptosis, genomic stability, cell metabolism, and tumor microenvironment.^[^ ^5^ ^]^ P53 inactivation is the most common tumor suppressor lesion and has been a major focus of oncology research, including in ICC.^[^ ^6^ ^]^ Adhesion G protein‐coupled receptor B1 (ADGRB1), a member of the G protein‐coupled receptors (GPCR) family, plays crucial roles in angiogenesis, cell adhesion, migration, and other processes relevant to tumor biology.^[^ ^7^ ^]^ Studies have demonstrated that ADGRB1 suppresses medulloblastoma formation by inhibiting mouse double minute 2 (MDM2)‐mediated p53 polyubiquitination.^[^ ^8^ ^]^ However, whether and how ADGRB1/p53 axis is regulated in ICC has not been explored yet.

CircRNAs are a subset of non‐coding RNAs generated from the back‐splicing of precursor mRNAs exons. Because of the covalently closed ring structure, circRNAs exhibit high stability and are resistant to exonuclease‐mediated degradation.^[^ ^9^ ^]^ CircRNAs exert diverse biological functions such as sponging miRNAs, interacting with RNA‐binding proteins (RBPs), regulating transcription and even serving as protein translation templates.^[^ ^10^, ^11^ ^]^ Dysregulated expression patterns of circRNAs have been observed in various cancer types, including ICC, suggesting their essential roles in tumor development and progression.^[^ ^12^, ^13^, ^14^ ^]^ However, the underlying mechanisms of circRNAs in ICC pathogenesis are still unclear, and interfering with circRNAs precisely at tumor sites remains a challenge.

In recent years, nanomedicine has emerged as a novel approach to cancer treatment. Nanoparticles are able to encapsulate and deliver siRNA, mRNA, DNA, and small molecular drugs with high stability and target affinity.^[^ ^15^, ^16^ ^]^ In order to explore the clinical translation of circRNAs in ICC treatment, we attempted to construct a lipid nanoparticle (LNP) system capable of effectively modulating circRNAs expression at the tumor site, thereby achieving anti‐tumor effects.

In this study, based on circRNA sequencing, we identified for the first time that circUGP2 (circBase ID: hsa_circ_0001020) functioned as a tumor suppressor in ICC progression, inhibiting the proliferation and metastasis of ICC cells both in vitro and in vivo. Furthermore, the results showed that circUGP2 interacted with purine‐rich element binding protein B (PURB), a transcription factor, in the promoter region of ADGRB1 to co‐activate ADGRB1 gene transcription and thereby activate ADGRB1/p53 tumor suppressor axis. Moreover, we found that circUGP2 could also regulate ADGRB1 expression through sponging miR‐3191‐5p. Based on these mechanisms, we constructed an LNP system to encapsulate and deliver circUGP2 plasmid to ICC cells, and its efficacy was validated through in vivo studies. Our findings provide a promising therapeutic strategy for ICC.

## 2 Results

### 2.1 Identification and Characteristics of circUGP2 in ICC

**Figure 1:**
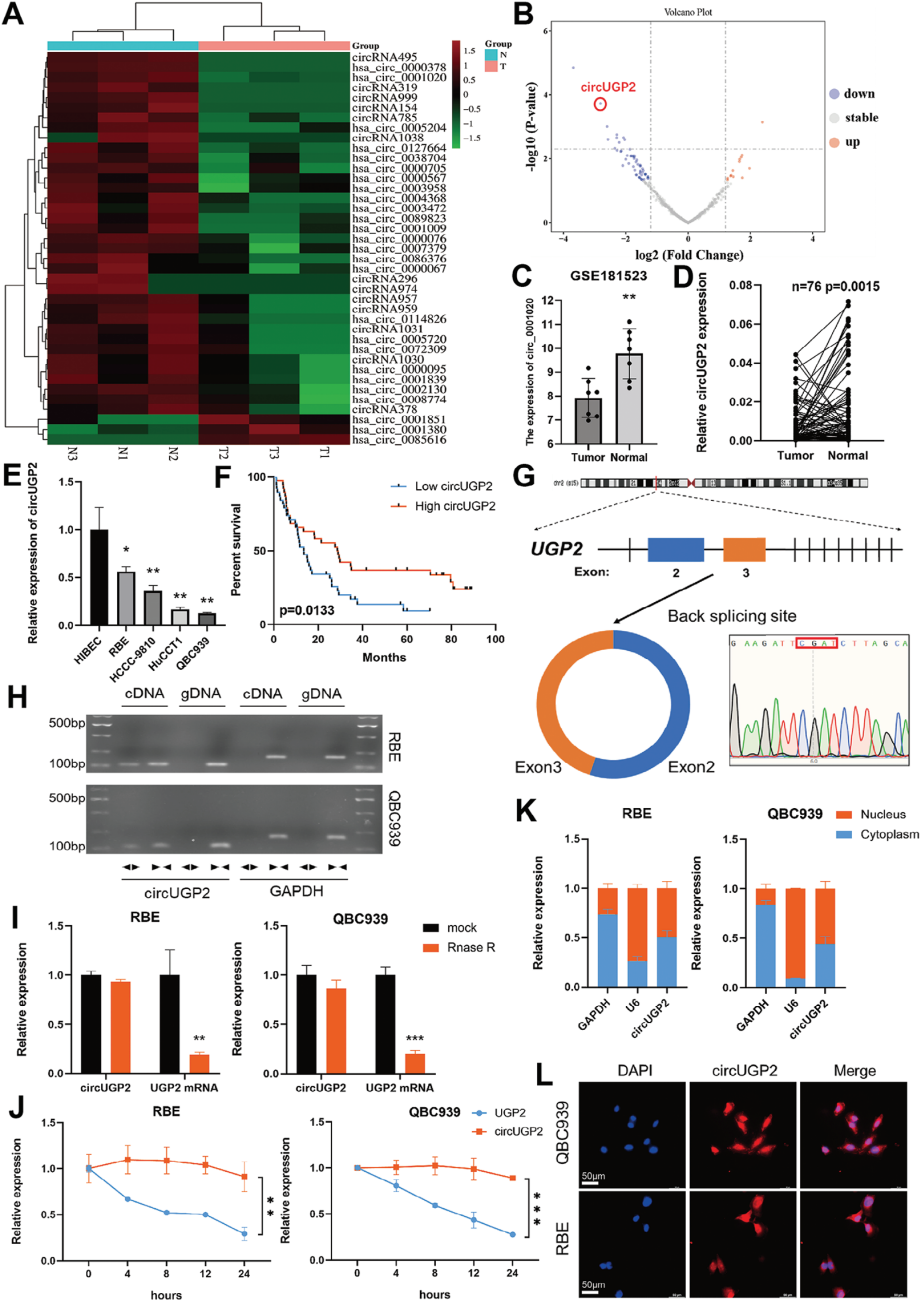
Identification and characteristics of circUGP2 in ICC. A) Heatmap of differentially expressed circRNAs in 3 paired ICC and normal tissues by circRNA‐seq. B) Volcano plot of differentially expressed circRNAs. C) Expression levels of circUGP2 in GSE181523 dataset. D) The levels of circUGP2 in 76 paired ICC and normal tissues detected by qRT‐PCR. E) The expression of circUGP2 in HiBEC, RBE, HCCC‐9810, HuCCT1, and QBC939 cell lines. (*n =* 3) F) Overall survival curve of 76 ICC patients stratified by the median expression of circUGP2. G) Schematic illustration of the genomic location and back splicing of circUGP2. H) Agarose gel electrophoresis analysis of the qRT–PCR products using divergent and convergent primers showing the amplification of circUGP2 from cDNA or genomic DNA (gDNA) in ICC cell lines. I) Expression levels of circUGP2 and UGP2 mRNA after RNase treatment. (*n =* 3) J) The expressions of circUGP2 and UGP2 mRNA were detected at the indicated time points after actinomycin D treatment. (*n =* 3) K) The subcellular localization of circUGP2 identified by nuclear and cytoplasmic fractionation assays. GAPDH and U6 were used as cytoplasmic and nuclear controls, respectively. (*n =* 3) L) FISH validated the subcellular location of circUGP2 in ICC cell lines. (scale bar, 50 µm) Data were present as mean ±SD. *p<0.05, **p<0.01, ***p<0.001. Paired D) or unpaired (C, E, I, J) Student's t‐test. Log‐rank test (F).

To screen for circRNAs involved in ICC development, 3 paired ICC and normal tissues were used for circRNA‐seq. The analysis unveiled 39 circRNAs with significant differential expression (*p<*0.05, log |FC|>2), among which hsa_circ_0 001020 (circUGP2) emerged as one of the most downregulated circRNAs in ICC (**Figure** **1**A,B). The down expression of circUGP2 was further validated in the gene expression profile (GSE181523) from the GEO database including 7 paired samples of ICC and normal tissues, in addition to our own set of 76 paired ICC tissues (Figure 1C,D). In cholangiocarcinoma cell lines, the expression of circUGP2 was also confirmed to be decreased compared with human intrahepatic biliary epithelial cell line (HiBEC), and we selected QBC939 and RBE cells for further study according to the results (Figure 1E). Additionally, the analysis of ICC patients’ data revealed a significant association between reduced levels of circUGP2 and unfavorable prognosis as well as advanced TNM stage (Figure 1F and **Table** **1**).

**Table 1:** Clinical characteristics of 76 ICC patients based on circUGP2 expression.

	circUGP2 expression		
Variables	Low (*n =* 38)	High (*n =* 38)	*p‐*Value
Gender			0.348
Male	25	21	
Female	13	17	
Age(years)			0.817
<60	21	22	
≥60	17	16	
Tumor differentiation			0.811
G1/G1‐G2/G2	13	14	
G2‐G3/G3	25	24	
MVI			0.150
No	28	33	
Yes	10	5	
Nerve invasion			0.387
No	29	32	
Yes	9	6	
HBsAg			0.813
Negative	24	23	
Positive	14	15	
T stage			0.017
T1	24	33	
T2‐T4	14	5	
N stage			0.006
N0	27	36	
N1	11	2	
TNM stage			0.004
I	19	31	
II‐III	19	7	

CircUGP2 derived from exons 2 and 3 of the UGP2 gene and its back‐splicing was verified by Sanger sequencing of qRT–PCR products using divergent primers (Figure 1G). Furthermore, agarose gel electrophoresis analysis of the qRT–PCR products amplified with convergent or divergent primers was performed. The results showed that circUGP2 was only amplified from cDNA samples using divergent primers, thus confirming the circular form of circUGP2 (Figure 1H). Moreover, the results of RNase R and actinomycin D treatment indicated that circUGP2 remained more stable than linear UGP2 (Figure 1I,J). In order to investigate the subcellular localization of circUGP2, nuclear and cytoplasmic fractionation assays and fluorescence in situ hybridization (FISH) were performed. The results showed the presence of circUGP2 transcripts in both the nucleus and cytoplasm of QBC939 and RBE cells (Figure 1K,L). Taken together, the above findings suggested that circUGP2 was downregulated in ICC and demonstrated the circular characteristics and subcellular localization of circUGP2.

### 2.2 CircUGP2 Inhibits ICC Progression In Vitro and In Vivo

**Figure 2:**
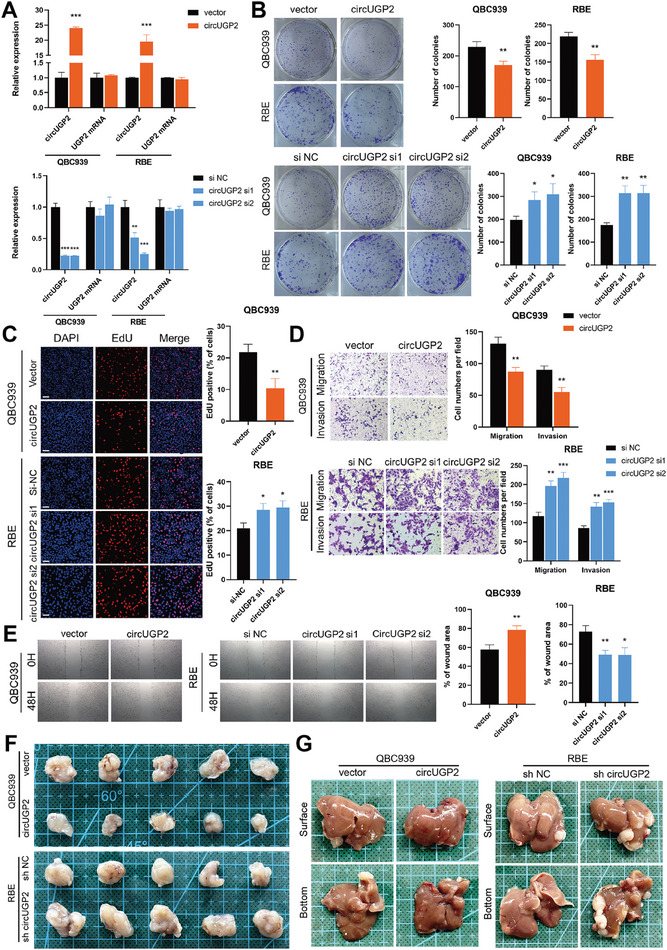
CircUGP2 inhibits the proliferation, migration, and invasion of ICC in vitro. A) The expressions of circUGP2 and UGP2 mRNA in ICC lines transfected with siRNAs or plasmid were detected by qRT‐PCR. (*n =* 3) B) Colony formation assays of QBC939 cells and RBE cells transfected with circUGP2 siRNAs or plasmid. (*n =* 3) C) EdU assays performed in circUGP2 knockdown or overexpression cells. (scale bar, 50 µm) (*n =* 3) D) Transwell assays were performed to evaluate the migration and invasion in QBC939 and RBE cells. (original magnification, 100X) (*n =* 3) E) Wound healing assays performed in these cell lines. (original magnification, 40X) (*n =* 3) F) Representative images of subcutaneous xenograft tumors in mice injected with stable circUGP2 knockdown or overexpression cells. (*n =* 5) G) Representative images of liver metastasis model. Data were present as mean ±SD. *p<0.05, **p<0.01, ***p<0.001. Unpaired Student's t‐test (A–E).

To investigate the biological function of circUGP2 in ICC, we used small interfering RNAs (siRNAs) targeting the back‐splicing junction for knockdown, as well as a plasmid for overexpression of circUGP2 in QBC939 and RBE cells. The efficiency of knockdown and overexpression of circUGP2 without affecting linear UGP2 mRNA expression was verified by qRT–PCR (**Figure** **2**A). As shown in Figure 2B, the results of clone formation assays demonstrated that circUGP2 knockdown promoted proliferation, while circUGP2 overexpression suppressed proliferation in QBC939 and RBE cells. These results were also verified by cell counting kit (CCK)−8 and EdU assays (Figure 2C; Figure S1A,B, Supporting Information). Furthermore, the abilities of migration and invasion were enhanced by circUGP2 knockdown and attenuated by circUGP2 overexpression, as evidenced by transwell assays and wound healing assays (Figure 2D,E; Figure S1C,D, Supporting Information).

To further examine the role of circUGP2 in ICC in vivo, QBC939 cells were stably transfected with lentiviruses for circUGP2 overexpression, while RBE cells were transfected with lentiviruses expressing short hairpin RNAs (shRNA), or the corresponding control vector. These cells were injected subcutaneously into nude mice. As shown in Figure 2F and Figure S1E (Supporting Information), the tumor growth was significantly inhibited by circUGP2 overexpression and facilitated by circUGP2 knockdown. These findings were further confirmed through Ki‐67 levels using immunohistochemistry (IHC) staining (Figure S1F, Supporting Information). To further assess the impact of circUGP2 on tumor metastasis, we established the liver metastasis model. The results showed that circUGP2 overexpression decreased the number of metastatic nodules in the liver, whereas circUGP2 knockdown increased it (Figure 2G; Figure S1G,H, Supporting Information). Altogether, these results demonstrated that circUGP2 suppressed ICC progression in vitro and in vivo.

### 2.3 CircUGP2 Activates the p53 Signaling Pathway in ICC

**Figure 3:**
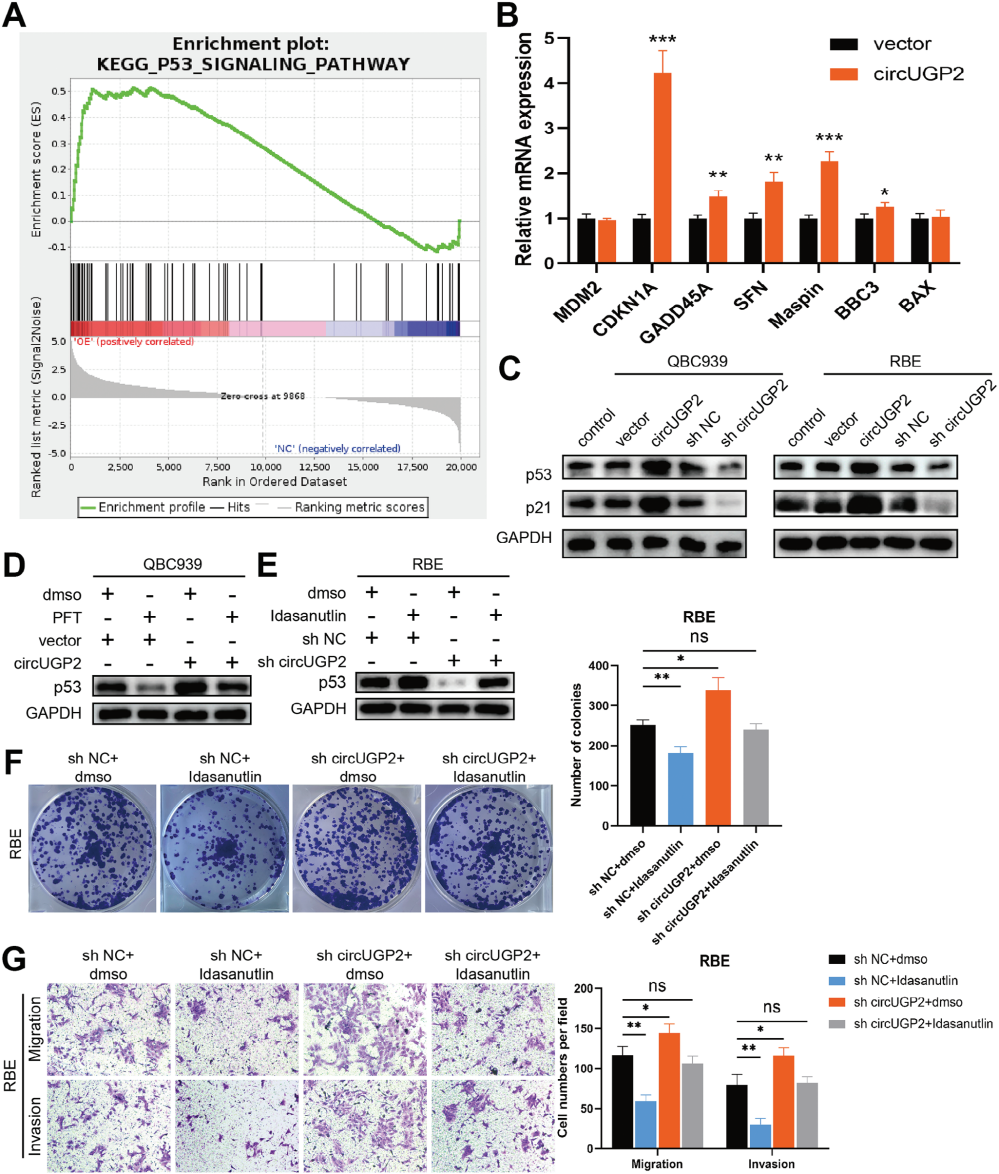
CircUGP2 activates the p53 signaling pathway in ICC. A) GSEA analysis for the circUGP2 overexpression group compared to the control group. B) qRT‐PCR analysis of p53 target gene expressions in circUGP2 overexpression and control group. (*n =* 3) C) Western blot analysis showed the levels of p53 and p21 in QBC939 and RBE cells. D) Protein levels of p53 in circUGP2 overexpression QBC939 cells treated with PFT and the corresponding control group. E) Protein levels of p53 in circUGP2 knockdown RBE cells treated with idasanutlin and the corresponding control group. F) Colony formation assays performed in RBE cells with the indicated treatment. (*n =* 3) G) Transwell assays performed in RBE cells. (original magnification, 100X) (*n =* 3) Data were present as mean ±SD. *p<0.05, **p<0.01, ***p<0.001. Unpaired Student's t‐test (B, F‐G).

To elucidate the molecular mechanisms of circUGP2 in suppressing ICC progression, we performed RNA‐seq and identified 882 differentially expressed genes in RBE cells overexpressing circUGP2 (*p<*0.05, log |FC|>2, Figure S2A, Supporting Information). The Kyoto Encyclopedia of Genes and Genomes (KEGG) pathway analysis showed that the PI3K‐Akt signaling pathway and p53 signaling pathway were enriched (Figure S2B, Supporting Information). However, the Gene Set Enrichment Analysis (GSEA) indicated that p53 signaling pathway was activated after circUGP2 overexpression, whereas PI3K‐Akt signaling pathway was not enriched (**Figure** **3**A; Figure S2C, Supporting Information). The qRT‐PCR analysis revealed the changed p53 target genes after circUGP2 overexpression (Figure 3B). Moreover, the results of the western blot demonstrated that p53 signaling pathway was activated by circUGP2 overexpression and inhibited by circUGP2 knockdown in QBC939 and RBE cells (Figure 3C), while PI3K‐Akt signaling pathway was unaffected (Figure S2D, Supporting Information).

Importantly, though p53 is the most frequently mutated gene in human cancers, it is noteworthy that QBC939 and RBE cell lines used in this study were reported to bear wild‐type (WT) p53 genes.^[^ ^17^, ^18^ ^]^ In order to verify whether the inhibitory role of circUGP2 in ICC progression was through regulating the p53 signaling pathway, we used pifithrin‐α hydrobromide (PFT), a p53 protein inhibitor,^[^ ^19^ ^]^ as well as idasanutlin, a p53‐MDM2 complex antagonist,^[^ ^20^ ^]^ to inhibit/activate the p53 signaling pathway (Figure 3D,E). Functionally, the results of clone formation assays and transwell assays showed that the adverse effect of circUGP2 knockdown was reversed by idasanutlin, while the effect of circUGP2 overexpression in inhibiting ICC progression was abrogated by PFT (Figure 3F,G; Figure S2E,F, Supporting Information). Taken together, these results suggested that circUGP2 inhibited ICC progression through activating the p53 signaling pathway.

### 2.4 CircUGP2 Interacts with PURB

**Figure 4:**
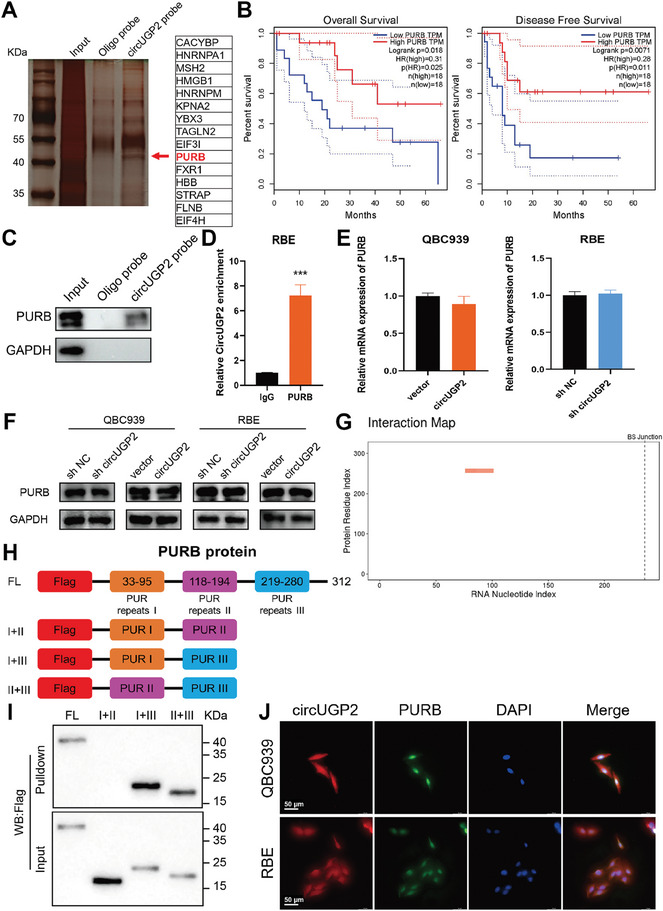
CircUGP2 interacts with PURB. A) CircUGP2 probe and oligo probe were used for RNA pull‐down assays in RBE lysates, followed by silver staining and MS analysis. 15 proteins with the highest scores identified in the MS analysis were listed. B) The overall survival curve and disease‐free survival curve of ICC patients are stratified by the median expression of PURB according to the GEPIA database. C) PURB was detected by western blot using proteins pulled down by a circUGP2 probe. D) CircUGP2 was detected through RIP assays using anti‐PURB or IgG antibodies. (*n =* 3) E) The mRNA levels of PURB were examined by qRT–PCR in circUGP2 overexpression and knockdown cells. (*n =* 3) F) PURB protein levels in circUGP2 overexpression and knockdown cells. G) The binding domain of PURB and circUGP2 was predicted by the catRAPID platform. H) Diagrams of the domain structure of Flag‐tagged PURB full‐length (FL) or truncation mutants. I) RNA pull‐down assays were performed in HEK‐293T cells transfected with the indicated vectors to identify the binding domain of PURB with circUGP2. J) The co‐localization of circUGP2 and PURB was detected by FISH assay and immunofluorescence. (scale bar, 50 µm) Data were present as mean ±SD. ***p<0.001. Unpaired Student's t‐test (D,E).

To delve into the regulatory role of circUGP2 in the p53 signaling pathway, a biotinylated circUGP2 probe was designed for RNA pull‐down in RBE lysates to identify the interacting proteins of circUGP2. The silver staining assay using proteins pulled down showed a specific band at ≈40 KD (**Figure** **4**A). Among 15 proteins with the highest scores identified in the mass spectrometry (MS) analysis, PURB became a focus because it was the only one that correlated with better prognosis in ICC patients according to the GEPIA platform^[^ ^21^ ^]^ (Figure 4A,B). The results of RNA pull‐down and RIP assays also confirmed that circUGP2 interacted with PURB in RBE cells, as determined by western blot and qRT–PCR (Figure 4C,D). Meanwhile, the clone formation assays and transwell assays indicated that PURB overexpression suppressed ICC progression and reversed the detrimental effect of circUGP2 knockdown (Figure S3A,B, Supporting Information). However, we observed that circUGP2 did not influence the mRNA and protein levels of PURB in QBC939 and RBE cells (Figure 4E,F).

The PURB protein is mainly composed of three domains of internal homology known as PUR repeats I, II, and III.^[^ ^22^ ^]^ Based on the prediction from catRAPID platform,^[^ ^23^ ^]^ the PUR repeats III was suggested to have the potential to interact with circUGP2 (Figure 4G). Therefore, several Flag‐tagged PURB truncation mutants were designed for RNA pull‐down assays and the results verified that PUR repeats III was the binding domain of PURB with circUGP2 (Figure 4H,I). FISH assay and immunofluorescence indicated the colocalization of circUGP2 and PURB in the nucleus of ICC cells (Figure 4J). Additionally, we examined the interaction between circUGP2 and PURB through structural prediction methods. The secondary structure of circUGP2 was predicted by the UNAfold platform^[^ ^24^ ^]^ and then submitted to 3dRNA platform^[^ ^25^ ^]^ to generate the tertiary structure. Subsequently, HDOCK platform^[^ ^26^ ^]^ was applied for the prediction of molecular docking between circUGP2 and PURB. The result revealed that they perfectly docked with each other (Figure S3C, Supporting Information). These results suggested that circUGP2 interacted with PURB during ICC progression.

### 2.5 CircUGP2 Functions as a Transcriptional co‐activator of PURB over the Expression of ADGRB1

**Figure 5:**
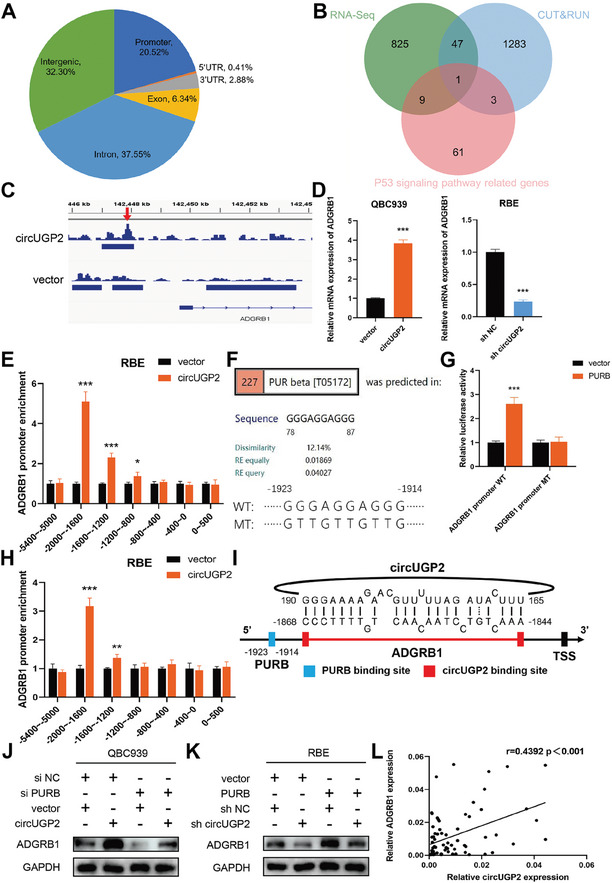
CircUGP2 functions as a transcriptional co‐activator of PURB over the expression of ADGRB1. A) Genome‐wide distribution of PURB‐binding regions in RBE cells using CUT&RUN. B) Venn diagram showed the overlap of the results of RNA‐seq, CUT&RUN, and p53 signaling pathway‐related genes from KEGG database. C) IGV tracks for PURB. D) The levels of ADGRB1 mRNA in circUGP2 overexpression and knockdown cells. (*n =* 3) E) CUT&RUN‐qPCR showed the binding site of PURB on ADGRB1 promoter. (*n =* 3) F) The specific binding site on the −2000 to −1600 bp region from the TSS of ADGRB1 promoter was predicted by PROMO platform. WT and MT ADGRB1 promoter plasmids for dual‐luciferase report assays were designed and synthesized based on the binding site. G) HEK‐293T cells were co‐transfected with WT or MT ADGRB1 promoter plasmid, combined with PURB overexpression plasmid or control vector for dual‐luciferase report assays to confirm the binding site of PURB on ADGRB1 promoter. (*n =* 3) H) ChIRP‐qPCR showed the binding site of circUGP2 on ADGRB1 promoter. (*n =* 3) I) Schematic illustration of the binding sites of circUGP2 and PURB on ADGRB1 promoter. J) Protein levels of ADGRB1 in QBC939 cells with circUGP2 overexpression and/or PURB knockdown, and the corresponding control group. K) Protein levels of ADGRB1 in RBE cells with circUGP2 knockdown and/or PURB overexpression, and the corresponding control group. L) Correlation analysis showed a positive relationship between circUGP2 and ADGRB1 in ICC tissues. Data were present as mean ±SD. *p<0.05, **p<0.01, ***p<0.001. Unpaired Student's t‐test (D, E, G, H). Pearson's correlation test L).

PURB has been reported to be a DNA and RNA binding protein capable of targeting the repeated purine‐rich element present in promoter regions, functioning as either a transcriptional activator or suppressor.^[^ ^27^, ^28^ ^]^ Therefore, we performed CUT&RUN in RBE cells with anti‐PURB antibody. The sequencing analysis revealed a broad distribution of PURB‐binding regions across the genome, with 20.52% of them identified as promoters (**Figure** **5**A). By overlapping the results of RNA‐seq and CUT&RUN, combined with p53 signaling pathway‐associated genes according to KEGG database, we identified ADGRB1 as the target for both circUGP2 and PURB in RBE cell (Figure 5B). The IGV tracks showed that the binding peaks of PURB were enriched in the promoter region of ADGRB1 gene and upregulated by circUGP2 overexpression (Figure 5C). Also, qRT–PCR demonstrated that the mRNA levels of ADGRB1 were upregulated by PURB or circUGP2 overexpression and downregulated by PURB or circUGP2 knockdown (Figure 5D; Figure S3D, Supporting Information). To further identify the binding site of PURB on ADGRB1 promoter, we performed CUT&RUN‐qPCR and found that PURB was mainly enriched in the −2000 to −1600 bp region from the transcription start site (TSS) of ADGRB1 promoter (Figure 5E). Subsequently, the specific binding site on this region was predicted by PROMO platform^[^ ^29^ ^]^ (Figure 5F), and further validated by dual‐luciferase reporter assays. Notably, PURB overexpression increased the luciferase activity in ADGRB1 promoter WT group, whereas no significant difference was observed in the mutant (MT) group (Figure 5G). Moreover, the knockdown of circUGP2 significantly decreased the luciferase activity while PURB overexpression restored the adverse effect (Figure S3E, Supporting Information). These findings suggested that PURB was a transcriptional activator of ADGRB1 and circUGP2 played a vital role in regulating this process.

Since the circUGP2 transcript was observed in the nucleus of QBC939 and RBE cells (Figure 1K,L), we next performed a ChIRP‐qPCR assay to examine whether circUGP2 regulated ADGRB1 transcription. The results revealed that circUGP2 was enriched in the same region of −2000 to −1600 bp from the TSS of ADGRB1 promoter as PURB (Figure 5H). The further analysis illustrated the binding sites of circUGP2 and PURB on the −2000 to −1600 bp region of ADGRB1 promoter (Figure 5I). Moreover, the results of western blot revealed that the effect of PURB overexpression in promoting ADGRB1 protein expression was abrogated by circUGP2 knockdown, while the adverse effect of PURB siRNA was restored by circUGP2 overexpression (Figure 5J,K). Additionally, qRT–PCR results showed a positive correlation between circUGP2 and ADGRB1 in ICC tissues (Figure 5L). Collectively, these findings indicated that circUGP2 functioned as a transcriptional co‐activator of PURB over the expression of ADGRB1.

### 2.6 CircUGP2 Upregulates ADGRB1 Expression Through Sponging miR‐3191‐5p

**Figure 6:**
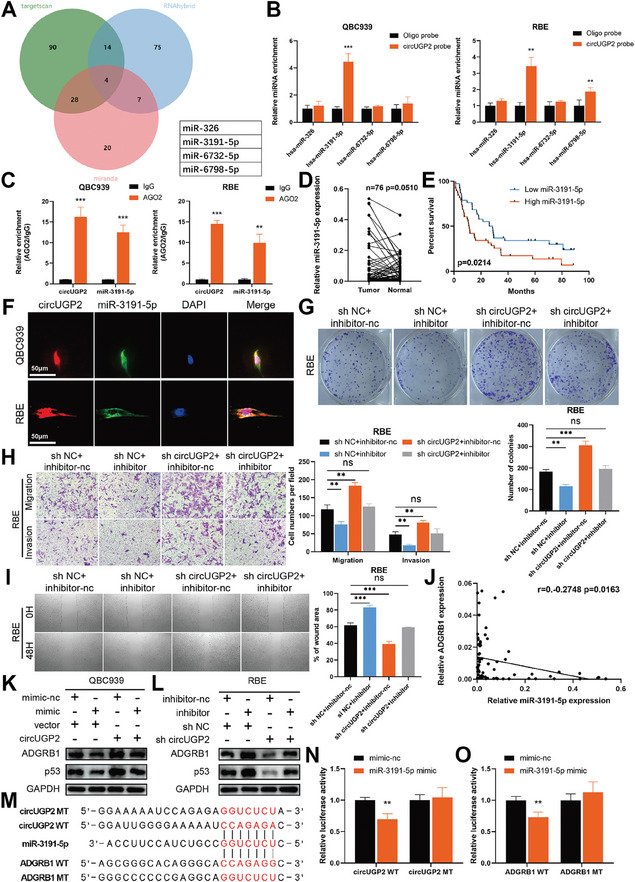
CircUGP2 upregulates ADGRB1 expression through sponging miR‐3191‐5p. A) 4 candidate miRNAs were listed by overlapping the prediction results of Targetscan, miRanda, and RNAhybrid. B) The enrichment of 4 candidate miRNAs were evaluated by RNA pull‐down assays using a circUGP2 probe in QBC939 and RBE cells. (*n =* 3) C) The interaction between circUGP2 and miR‐3191‐5p was determined by RIP assays using anti‐AGO2 or IgG antibodies. (*n =* 3) D) The expressions of miR‐3191‐5p in 76 paired ICC and normal tissues were detected by qRT‐PCR. E) Overall survival curve of 76 ICC patients stratified by the median expression of miR‐3191‐5p. F) The colocalization of circUGP2 and miR‐3191‐5p was detected by FISH assay. (scale bar, 50 µm) G) Colony formation assays were performed in circUGP2 knockdown RBE cells treated with miR‐3191‐5p inhibitor, as well as the corresponding control groups. (*n =* 3) H) Transwell assays performed in RBE cells. (original magnification, 100X) (*n =* 3) I) Wound healing assays performed in RBE cells with the indicated treatment. (original magnification, 40X) (*n =* 3) J) Correlation analysis showed a negative relationship between miR‐3191‐5p and ADGRB1 in ICC tissues. K,L) ADGRB1 and p53 expression were determined by western blot in QBC939 and RBE cells with the indicated treatment. M) The binding sites between miR‐3191‐5p and circUGP2 or ADGRB1 3′‐UTR were predicted by RNAhybrid to construct the wild‐type (WT) and mutant (MT) dual‐luciferase reporter plasmids. N,O) The interactions between miR‐3191‐5p and circUGP2 or ADGRB1 were confirmed by dual‐luciferase report assays. (*n =* 3) Data were present as mean ±SD. **p<0.01, ***p<0.001. Paired D) or unpaired (B,C,G–I,N,O) Student's t‐test. Log‐rank test E). Pearson's correlation test J).

CircRNAs in the cytoplasm tend to act as miRNA sponges.^[^ ^11^ ^]^ Considering the circUGP2 transcript was also observed in the cytoplasm of QBC939 and RBE cells (Figure 1K,L), we assumed that circUGP2 could also sponge miRNA in ICC. Therefore, 4 candidate miRNAs (miR‐326, miR‐3191‐5p, miR‐6732‐5p, and miR‐6798‐5p) were identified by overlapping the miRNA target prediction of Targetscan, miRanda and RNAhybrid^[^ ^30^, ^31^, ^32^ ^]^ (**Figure** **6**A). Then we performed RNA pull‐down assays using a biotinylated circUGP2 probe and the results revealed that miR‐3191‐5p was enriched in both QBC939 and RBE cells (Figure 6B). The results of RIP assays also showed that both circUGP2 and miR‐3191‐5p were immunoprecipitated by anti‐Argonaute2 (AGO2) antibodies (Figure 6C). Moreover, miR‐3191‐5p was confirmed to be upregulated in ICC tissues (*p =* 0.051) and a decreased level of miR‐3191‐5p was demonstrated to be associated with improved survival (Figure 6D,E). The results of the FISH assay indicated the colocalization of circUGP2 and miR‐3191‐5p in the cytoplasm of QBC939 and RBE cells (Figure 6F). To explore the role of miR‐3191‐5p in ICC progression, miR‐3191‐5p inhibitor or mimic was transfected in RBE or QBC939 cells. The results of CCK‐8, clone formation, transwell, and wound healing assays showed that miR‐3191‐5p inhibitor suppressed ICC progression, whereas miR‐3191‐5p mimic promoted it. Notably, these effects were subsequently counteracted by circUGP2 knockdown or overexpression (Figure 6G–I; Figure S4A–D, Supporting Information).

Interestingly, we noticed that ADGRB1 expression was negatively correlated with miR‐3191‐5p (Figure 6J), and ADGRB1 was predicted by miRWalk^[^ ^33^ ^]^ as a possible target gene of miR‐3191‐5p. Meanwhile, the protein levels of ADGRB1 and p53 were downregulated by miR‐3191‐5p mimic and upregulated by miR‐3191‐5p inhibitor, which could be reversed by circUGP2 overexpression or knockdown as well (Figure 6K,L). Hence, it was hypothesized that ADGRB1 could also be regulated by miR‐3191‐5p in ICC. Subsequently, the binding sites between miR‐3191‐5p and circUGP2 or ADGRB1 3′‐untranslated region (3′‐UTR) were predicted by RNAhybrid to construct the mutant dual‐luciferase reporter plasmids (Figure 6M). The results of dual‐luciferase report assays showed that co‐transfection with miR‐3191‐5p mimic significantly decreased the luciferase activity in the WT circUGP2 or ADGRB1 3′‐UTR group, while no difference was observed in the MT group (Figure 6N,O). Moreover, circUGP2 overexpression restored the luciferase activity decreased by miR‐3191‐5p mimic (Figure S4E, Supporting Information). Collectively, these results proved that circUGP2 could also upregulate ADGRB1 expression through sponging miR‐3191‐5p.

### 2.7 CircUGP2 Activates ADGRB1/p53 Axis to Suppress ICC Progression

**Figure 7:**
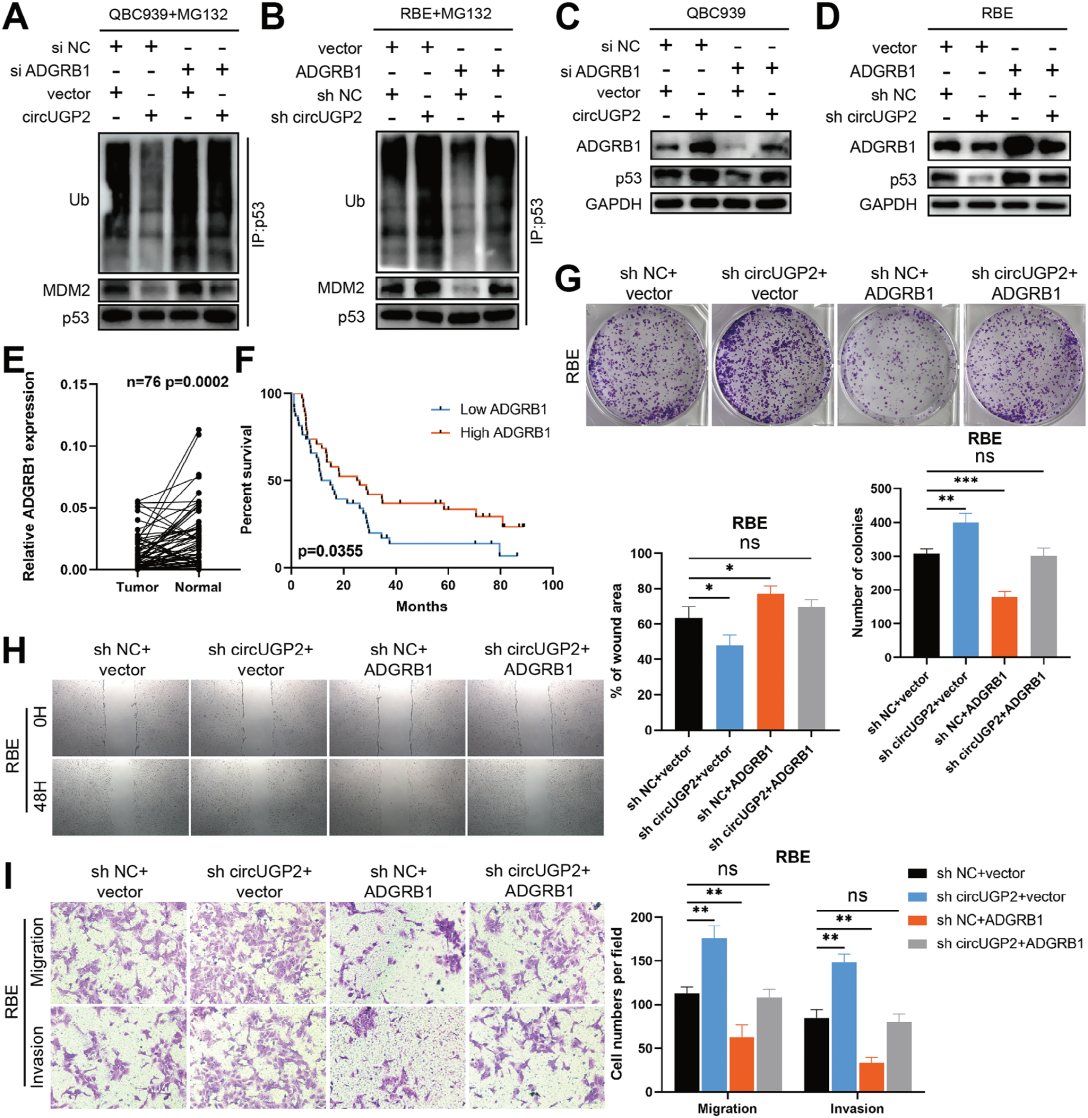
CircUGP2 activates ADGRB1/p53 axis to suppress ICC progression. A) Co‐IP assays were performed to determine the MDM2‐mediated p53 polyubiquitination levels in QBC939 cells with circUGP2 overexpression and/or ADGRB1 knockdown, and the corresponding control group. B) The MDM2‐mediated p53 polyubiquitination levels in RBE cells were detected by Co‐IP assays. C,D) Protein levels of ADGRB1 and p53 in these cells were examined by western blot. E) ADGRB1 mRNA levels in 76 paired ICC and normal tissues. F) Overall survival curve of 76 ICC patients stratified by the median expression of ADGRB1. G) Colony formation assays performed in RBE cells with the indicated treatment. (*n =* 3) H) Wound healing assays performed in RBE cells. (original magnification, 40X) (*n =* 3) I) Transwell assays were performed to evaluate the migration and invasion in RBE cells. (original magnification, 100X) (*n =* 3) Data were present as mean ±SD. *p<0.05, **p<0.01, ***p<0.001. Paired E) or unpaired (G–I) Student's t‐test. Log‐rank test (F).

ADGRB1 has been reported to prevent MDM2‐mediated p53 polyubiquitination, consequently activating p53 signaling pathway in medulloblastoma.^[^ ^8^ ^]^ In order to investigate the role of ADGRB1 in ICC via circUGP2, we performed coimmunoprecipitation (co‐IP) assays. The findings showed that ADGRB1 siRNA promoted the MDM2‐mediated p53 polyubiquitination, which was counteracted by circUGP2 overexpression (**Figure** **7**A). Conversely, ADGRB1 overexpression exhibited the opposite effect, which was abrogated by circUGP2 knockdown (Figure 7B). As expected, these effects were confirmed by western blot (Figure 7C,D). After overexpressing MDM2, the upregulated p53 polyubiquitination level was restored by circUGP2 overexpression (Figure S5A, Supporting Information). Moreover, qRT–PCR analysis revealed that TP53 mRNA level was not affected by ADGRB1 (Figure S5B, Supporting Information). These findings demonstrated that circUGP2 regulated p53 protein levels through upregulating ADGRB1 to prevent MDM2‐mediated p53 polyubiquitination in ICC cells.

Additionally, the down expression of ADGRB1 mRNA levels was validated in our 76 paired ICC tissues, which was associated with a negative outcome (Figure 7E,F). Furthermore, in vitro studies of clone formation assays, transwell assays, and wound healing assays showed that the negative effect of circUGP2 knockdown could be rescued by ADGRB1 overexpression, while the inhibiting role of circUGP2 overexpression was counteracted by ADGRB1 knockdown (Figure 7G–I; Figure S5D–F, Supporting Information). In summary, these findings confirmed that circUGP2 suppressed ICC progression by activating the ADGRB1/p53 axis.

### 2.8 Therapeutic Strategy Using LNP‐encapsulated circUGP2 Plasmid Reveals Anti‐Tumor Effects

**Figure 8:**
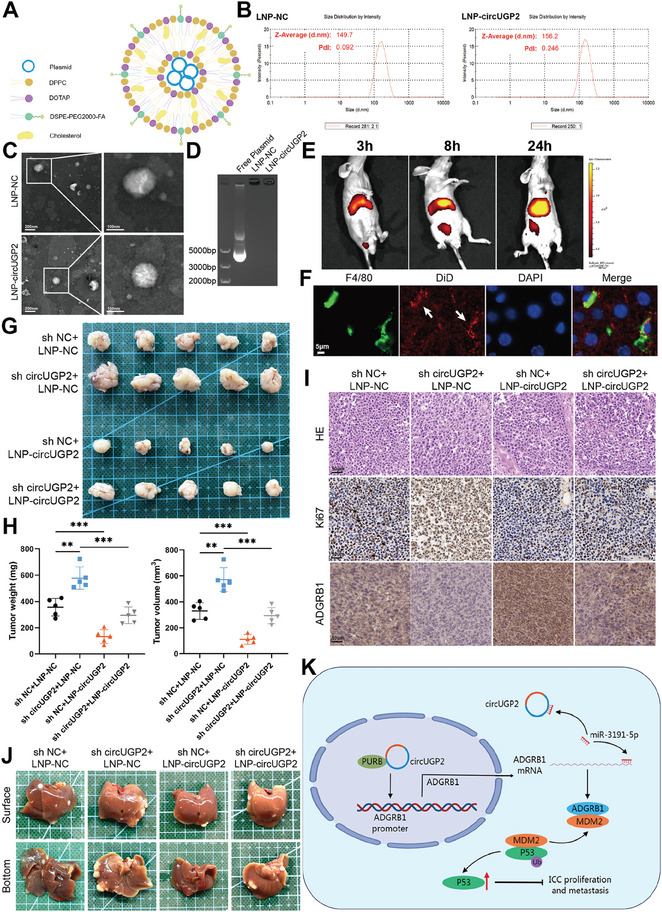
Therapeutic strategy using LNP‐encapsulated circUGP2 plasmid reveals anti‐tumor effects. A) Schematic illustration of the LNP system that encapsulated circUGP2 plasmid. B) The diameter and polydispersity index (PdI) of LNP‐NC and LNP‐circUGP2 were detected by DLS. C) Representative TEM images of LNP‐NC and LNP‐circUGP2. (scale bar, left: 200 nm, right: 100 nm) D) The agarose gel electrophoresis analysis of free plasmid, LNP‐NC, and LNP‐circUGP2. E) Fluorescence images showed the biodistribution of DiD‐labeled LNP in subcutaneous tumor‐bearing mice at the indicated times. F) Immunofluorescence experiments of liver sections from mice injected with LNPs. The Kupffer cells were stained with F4/80 (green). LNPs were labeled with DiD (red). All nuclei were stained with DAPI (blue). (scale bar, 5 µm) G) Representative images of subcutaneous xenograft tumors in mice injected with circUGP2 knockdown RBE cells and treated with LNPs. (*n =* 5) H) Comparisons of tumor weights and volumes between groups with the indicated treatment. (*n =* 5) I) Representative images of H&E, Ki‐67, and ADGRB1 staining of the subcutaneous xenograft tumors. (scale bar, 50 µm) J) Representative images of liver metastasis model. K) Schematic diagram illustrating the mechanism by which circUGP2 suppressed ICC progression. Data were present as mean ±SD. **p<0.01, ***p<0.001. Unpaired Student's t‐test (H).

LNP delivery strategy is a revolutionary therapeutic development, enabling the delivery of siRNA, mRNA, DNA, and small molecular drugs.^[^ ^15^ ^]^ The folate receptor (FR) is upregulated in many human tumors and recently a study using a folate‐labeled LNP delivery system targeting ICC showed superior anti‐tumor effects.^[^ ^34^, ^35^ ^]^ The result of qRT–PCR verified that FR alpha (FOLR1) was overexpressed in RBE cells in comparison to HiBEC cells (Figure S6A, Supporting Information). Therefore, we established a similar LNP delivery system to encapsulate circUGP2 plasmid aimed to target ICC cells, using DOTAP, DPPC, folate‐conjugated DSPE‐PEG2000, and cholesterol (**Figure** **8**A). The results of dynamic light scattering (DLS) revealed the diameters with an average of 149.7 nm (LNP‐NC) and 156.2 nm (LNP‐circUGP2) (Figure 8B). The images of LNPs were shown by transmission electron microscopy (TEM) (Figure 8C). Meanwhile, LNP‐NC and LNP‐circUGP2 did not exhibit electrophoretic shift compared to free plasmid as shown by the agarose gel electrophoresis, indicating that circUGP2 plasmid was successfully encapsulated into LNPs (Figure 8D). In order to evaluate the targeting ability and delivery efficiency of LNPs, fluorescence imaging was performed on subcutaneous tumor‐bearing mice after being injected with DiD‐labeled LNPs through the tail vein. As shown in Figure 8E and Figure S6B (Supporting Information), the fluorescence signals were accumulated in the tumor site, lasting at least 24 h. As nanoparticles are often taken up by Kupffer cells in the liver, the immunofluorescence experiments of liver sections showed that LNPs were phagocytosed by the Kupffer cells (Figure 8F). However, the results of qRT–PCR demonstrated that FOLR1 was overexpressed in RBE cells than mice primary hepatocytes and Kupffer cells (Figure S6A, Supporting Information), and study has reported that polyethylene glycol (PEG) and folate could reduce phagocytosis by macrophages and effectively target ICC cells.^[^ ^35^ ^]^

To further explore the anti‐tumor effects of LNP‐circUGP2, a subcutaneous tumor xenograft model was established using RBE cells with stable circUGP2 knockdown, and LNP‐NC or LNP‐circUGP2 was injected through the tail vein twice weekly for 4 weeks. The results showed that LNP‐circUGP2 treatment upregulated circUGP2 levels and activated the p53 signaling pathway in the subcutaneous tumors (Figure S6C–E, Supporting Information), resulting in the suppression of tumor growth and the reversal of the negative impacts associated with circUGP2 knockdown (Figure 8G,H). These results were further confirmed by IHC staining as Ki‐67 levels were downregulated and ADGRB1 levels were upregulated after LNP‐circUGP2 treatment (Figure 8I). Moreover, in the liver metastasis model, LNP‐circUGP2 treatment reduced liver metastatic nodules (Figure 8J; Figure S6F, Supporting Information). As for the biosafety of LNPs, the body weight and H&E sections of major organs of the subcutaneous tumor‐bearing mice showed no obvious abnormalities after LNP treatment (Figure S6G,H, Supporting Information). Also, there were no significant changes in serum ALT, AST, and CREA levels (Figure S6I, Supporting Information). Therefore, our findings provided a promising and safe strategy using LNP‐encapsulated circUGP2 plasmid for ICC treatment.

## 3 Discussion

Despite recent advancements in the study and treatment of ICC, the prognosis remains unfavorable. The development of sequencing technology has enabled the identification of circRNAs' roles in various tumors, exerting multiple cellular functions. In this paper, we identified a downregulated circRNA‐circUGP2 in ICC tissues through circRNA‐seq and GSE181523 dataset. The expression of circUGP2 was correlated with patients’ prognosis and its inhibiting role in ICC was further verified through in vitro and in vivo study.

Currently, the mechanism of circRNAs interacting with RBPs is becoming popular. By interacting with different proteins, circRNAs can sequester them or activate their functions as scaffolds.^[^ ^10^ ^]^ In the current study, the results of RNA pull‐down assay and MS indicated the interaction between circUGP2 and PURB. Moreover, by performing RNA pull‐down assays using Flag‐tagged PURB truncation mutants, we proved that circUGP2 specifically interacted with the PUR repeats III domain of PURB. PURB is a highly conserved protein, able to bind to the repeated purine‐rich element in promoter regions. Its dual roles as both a transcriptional activator and suppressor have been reported.^[^ ^27^, ^28^ ^]^ In this study, the results of RNA‐seq and CUT&RUN revealed their role in initiating ADGRB1 transcription. By co‐activating transcription factors, circRNAs can modulate transcription. For example, circIPO11 is reported to recruit TOP1 to the GLI1 promoter and circIKBKB could raise NF‐κB to the promoters of M‐CSF and GM‐CSF, thereby triggering transcription and activating downstream signaling.^[^ ^36^, ^37^ ^]^ The results of the ChIRP assay in our study displayed the interaction between circUGP2 and ADGRB1 promoter, indicating the co‐activating role of circUGP2 and PURB in ADGRB1 transcription.

ADGRB1, also known as brain angiogenesis inhibitor 1 (BAI1), belongs to GPCR and is involved in multiple cancer development.^[^ ^8^, ^38^, ^39^, ^40^, ^41^ ^]^ ADGRB1 has been reported to have antiangiogenic effects, and suppressing ADGRB1 by MBD2 leads to tumor growth in glioblastoma.^[^ ^40^, ^42^ ^]^ In medulloblastoma and T‐cell acute lymphoblastic leukemia, the interaction between ADGRB1 and MDM2 prevents MDM2‐mediated p53 polyubiquitination,^[^ ^8^, ^41^ ^]^ which is consistent with our findings in ICC.

The tumor suppressor p53 is a transcription factor regulating multiple genes and cellular functions. In ICC, the inactivation of p53 has been implicated in various studies.^[^ ^43^ ^]^ It has been reported that miR‐191 could downregulate TET1 levels, leading to p53 gene TSS staying methylated and thereby promoting ICC progression.^[^ ^17^ ^]^ Given that p53 is the most frequently mutated gene in human cancers, targeting p53 has emerged as an attractive approach for anti‐tumor therapy. In particular, blocking p53‐MDM2 interaction has been a hotspot. The binding between p53 and MDM2 protein promotes the transfer of p53 protein from the nucleus to the cytoplasm, inactivates p53 transcriptional function, and enhances p53 polyubiquitination.^[^ ^44^ ^]^ In our study, the p53 signaling was activated by circUGP2 through upregulating ADGRB1 expression to prevent MDM2‐mediated p53 polyubiquitination.

Additionally, competing endogenous RNA (ceRNA) network is another hotspot in circRNAs studies. CircRNAs located in the cytoplasm can act as sponges for miRNAs, inhibiting their activity and consequently upregulating the downstream mRNA expression.^[^ ^45^ ^]^ In ICC, circCCAC1 has been reported to promote tumor progression by sponging miR‐514a‐5p to upregulate YY1.^[^ ^46^ ^]^ Chen. et al. also reported that circACTN4 could sponge miR‐424‐5p to upregulate YAP1, leading to ICC progression.^[^ ^14^ ^]^ In the current study, circUGP2 was observed in both the nucleus and cytoplasm of ICC cells, and further investigations revealed that ADGRB1 could also be upregulated by circUGP2 through sponging miR‐3191‐5p.

Although circRNAs have been extensively studied and have shown great potential for cancer therapy, interfering with circRNAs at tumor sites remains a challenge. LNP delivery strategy is a revolutionary clinical translation of gene therapies with the advantages of ease of manufacture, reduced immune responses, high stability in the circulation, and increased target affinity.^[^ ^15^, ^16^ ^]^ LNP is able to deliver siRNA, mRNA, DNA, and small molecular drugs. Plasmid DNA encapsulated in LNP has also been reported to exhibit in vivo activity. Zhou. et al. constructed LNPs encapsulating plasmids loading human CAR gene and IL‐6 shRNA, which successfully established CAR‐T cells with IL‐6 knockdown in mice.^[^ ^47^ ^]^ Recently, a circRNA study utilized a folate‐labeled LNP system co‐loaded with siRNA and paclitaxel to target ICC, showing superior anti‐tumor effects.^[^ ^35^ ^]^ Based on these studies, we established an LNP system to encapsulate circUGP2 plasmid using folate‐conjugated DSPE‐PEG2000, which successfully targeted the tumor site. Notably, in vivo studies verified the significant anti‐tumor effects of LNP‐circUGP2.

In summary, the present study identified circUGP2 as a tumor suppressor in ICC by interacting with PURB to co‐activate ADGRB1 transcription, as well as sponging miR‐3191‐5p to upregulate ADGRB1 expression, leading to the activation of p53 signaling pathway. Moreover, the application of the LNP system to deliver circUGP2 plasmid to the tumor site provides a promising translational strategy for ICC treatment.

## 4 Experimental Section

### Human Tissue Samples

From Jan. 2011 to Apr. 2015, a total of 76 paired ICC and normal tissues were obtained from the resected specimens of ICC patients that underwent surgery at The First Affiliated Hospital of Nanjing Medicine University. All samples were immediately frozen in liquid nitrogen and stored at −80 °C. This study was approved by the Ethics Committee of The Affiliated Hospital of Nanjing Medical University and in accordance with the Declaration of Helsinki. Each patient had signed the informed consent ahead of participation in this study. Samples inclusion criteria: i) Confirmation of tissues as ICC by two independent pathologists, ii) Absence of radiotherapy, chemotherapy, or other related therapy before surgery, iii) Postoperative survival time exceeding one month. Exclusion criteria: i) Presence of radiotherapy, chemotherapy, or other related therapy before surgery, ii) Presence of other malignant diseases, iii) Postoperational survival time less than one month.

### RNA‐seq and Data Analysis

The circRNA‐seq of 3 paired ICC tissues was conducted by LC‐Bio Technologies (China). Limma R package was used for differential expression analysis in R 4.2.1 with R studio. The expressions of screened circRNAs were verified in the profile GSE181523 from the GEO database (https://www.ncbi.nlm.nih.gov/geo/), including 7 paired samples of ICC and normal tissues. The RNA‐seq of RBE cells with circUGP2 overexpression and the corresponding control group was conducted by Biomarker Technologies (China). Total RNA was extracted using TRlzol Reagent (Life Technologies, USA), followed by the generation of sequencing libraries using the Ultima Dual‐mode mRNA Library Prep Kit for Illumina (Yeasen Biotechnology, China) Subsequently, the libraries were sequenced to a read depth of 20 million reads per sample on Illumina NovaSeq6000 platforms (Illumina, USA). Reads containing adapters, poly‐N, and low‐quality reads in the raw data were excluded. The FASTQ reads were aligned to the GRCh38 reference human genome. Differential expression analysis was conducted using the DEseq2 R package in R 4.2.1 with R Studio.

### Quantitative Real‐time PCR (qRT‐PCR)

Total RNA from tissues or cells was extracted by RNA‐Quick Purification Kit (Yishan, China) and reverse‐transcribed using a cDNA reverse transcription kit (Vazyme, China) according to the manufacturer's instructions. qRT‐PCR was then performed with SYBR Green (Vazyme, China) using the 7900HT Fast Real‐Time PCR System (Applied Biosystems, USA). The relative mRNA levels were normalized against GAPDH or U6 mRNA levels. The primer sequences were listed in Table S1 (Supporting Information).

### Fluorescence In Situ Hybridization (FISH)

Fluorescently labeled circUGP2 and miR‐3191‐5p probes were designed and synthesized by RiboBio Company (China). FISH was performed using a FISH Kit (RiboBio Company, China). Following the procedures of fixation, permeabilization, and prehybridization, cells were incubated with a FISH probe overnight at 37 °C and then washed with 4 × SSC, 2 × SSC, and 1 × SSC at 42 °C gradually. Nuclei were stained with DAPI. The samples were photographed by THUNDER Imaging Systems (Leica, Germany).

### Actinomycin D Assay

QBC939 and RBE cells were treated with 2 µg mL^−1^ actinomycin D (MCE, USA) for the indicated times. The expressions of circUGP2 and UGP2 mRNA were detected by qRT–PCR.

### RNase R treatment

Total RNA was extracted from QBC939 and RBE cells and treated with 2U/µg of RNase R (Geenseed, China) or RNase‐free water. The expressions of circUGP2 and UGP2 mRNA were detected by qRT–PCR.

### Agarose Gel Electrophoresis

Agarose gels (2%) were made by adding 2 g agarose into 100 mL TAE running buffer and stained with Goldview (Coolaber, China). Nucleic acid samples were loaded into each well and run at 80 V. Gel images were taken by FUSION FX Imaging System (Vilber GmbH, France).

### Nuclear and Cytoplasmic Fractionation

Nuclear and cytoplasmic fractions were isolated by PARIS kit (Thermo, USA). Briefly, QBC939 and RBE cells were lysed by Cell Fraction Buffer and then centrifuged at 500 g for 3 min at 4 °C. The supernatant was the cytoplasmic fraction and the nuclear fraction was extracted by incubating the pellet with Cell Disruption Buffer. The products were mixed with Lysis/Binding Solution and absolute ethanol and then added to the Filter Cartridge. After being washed 3 times, the RNA was eluted.

### Cell culture and Transfection

HiBEC, RBE, HCCC9810, HuCCT1, and QBC939 cell lines were purchased from the Cell Bank of the Chinese Academy of Sciences (China). All cells were cultured in DMEM medium (Gibco, USA) with 10% fetal bovine serum (Gibco, USA) and 1% penicillin‐streptomycin (Gibco, USA), and maintained in an incubator supplemented with 5% CO2 at 37 °C. HEK‐293T cells were purchased from ATCC and cultured with the same condition.

All siRNAs were purchased from GenePharma (China) and plasmids were synthesized by Corues Biotechnology (China). The siRNA sequences were listed in Table S1 (Supporting Information). For circUGP2 overexpression plasmid, the full length of circUGP2 was cloned into the EcoRI/BamHI‐digested pCMV3‐SV40‐GFP‐Puro‐LB‐RB vector (Corues Biotechnology, China). For cell transfection, LipofectamineTM 2000 (Invitrogen, US) was used according to the manufacturer's protocol. For stable transfected cell lines, lentiviruses with circUGP2 overexpression or shRNA expression and their corresponding control vector were synthesized by GenePharma (China). The full length of circUGP2 was amplified and PCR product was cloned into the NotI/BamHI‐digested LV5(EF‐1a/GFP&Puro) vector (GenePharma, China) using the ClonExpress Entry One Step Cloning Kit (Vazyme, China). The titration was 1 × 10^9^ TU mL^−1^. The shRNA of circUGP2 was cloned into the LV‐3(pGLVH1/GFP+Puro) vector (GenePharma, China). The titration was 1 × 10^8^ TU mL^−1^. The shRNA sequence was listed in Table S1 (Supporting Information). Stable cell lines were screened out with 10 µg mL^−1^ puromycin for 3 consecutive days following 72 h of transfection. For p53 signaling inhibition and activation, QBC939 cells were treated with 10 µm PFT (MCE, USA) for 1 h, and RBE cells were treated with 200 nM idasanutlin (MCE, USA) for 48 h.

### Cell counting kit (CCK)−8 Assay

One thousand cells per well were seeded into the 96‐well plates and added with 10% CCK‐8 solution (Dojindo, Japan) each day. After incubation in the dark for 2 h, the 96‐well plates were sent to absorbance measurement at 450 nm by Elx800 microplate reader (Bio‐Tek, USA).

### 5‐Ethynyl‐20‐deoxyuridine (EdU) Assay

EdU cell proliferation kit (Beyotime, China) was used for EdU assays. Briefly, cells were treated with 10 µm EdU for 2 h and fixed with 4% paraformaldehyde, followed by permeabilization by 0.3% Triton for 15 min. After that, cells were stained using the Click Reaction Buffer containing Azide 555 for 30 min and DAPI for 10 min. The images were taken by THUNDER Imaging Systems (Leica, Germany).

### Clone Formation Assay

Cells were seeded into the 6‐well plates (1000 cells per well for QBC939, 1500 cells/well for RBE) and cultured for 10 days. Then the cells were fixed with 4% paraformaldehyde and stained using a Crystal Violet Staining Solution (Beyotime, China).

### Wound Healing Assay

Cells were seeded into the 6‐well plates. A 200‐µL pipette tip was applied to make a straight wound when the cell density reached 90%−100%. The images were taken by an inverted microscope (ZEISS, Germany).

### Transwell Assay

Cells (2 × 10^4^) cultured in serum‐free medium were seeded into the upper chamber with (invasion) or without (migration) Matrigel (Corning, USA). The lower chamber was added with a medium containing 20% fetal bovine serum. After 2 days, cells were fixed and stained with a Crystal Violet Staining Solution (Beyotime, China). After removing the remaining cells in the upper chamber surface, images of cells on the bottom surface were taken by a light microscope (Olympus, Japan).

### Western Blot

Western blot was performed routinely. Cells or tissue samples were lysed using RIPA Lysis Buffer (Beyotime, China). The proteins obtained were separated by SDS‐PAGE and transferred to polyvinylidene fluoride membranes, which were subsequently blocked with the QuickBlock Blocking Buffer (Beyotime, China). The membranes were incubated with primary antibodies at 4 °C overnight and then incubated with the secondary antibody at room temperature for 2 h the next day. Finally, the proteins were detected by the FUSION FX Imaging System (Vilber GmbH, France) using an Ultra‐sensitive ECL chemiluminescent substrate (Biosharp, China). The relative protein levels were normalized against GAPDH protein levels. The antibodies were listed in Table S2 (Supporting Information).

### RNA Pull‐Down Assay

Biotinylated circUGP2 probe and oligo probe were designed by RiboBio Company (China). The probe sequences were listed in Table S1 (Supporting Information). After incubating the probes with Streptavidin Coated Beads (BEAVER biomedical, China), the lysates of RBE cells were incubated with the beads at 4 °C overnight. As a result, miRNAs were pulled down and extracted, detected by qRT–PCR. The pulled‐down proteins were performed with silver staining assay, mass spectrometry (MS) analysis, and western blot.

### RNA Immunoprecipitation (RIP) Assay

RIP assays were performed using the RNA Immunoprecipitation Kit (Geneseed, China) according to the manufacturer's instructions. Firstly, cells were lysed by 1 × Buffer A (containing 1% protease inhibitor and RNase inhibitor). 100 µL protein A+G beads per tube were pretreated with 0.5 mL 1 × Buffer A and 10 µL Buffer D at 4 °C for 30 min and then washed with 1 × Buffer A twice. Subsequently, the protein A+G beads were added with 1 mL 1 × Buffer A and incubated with anti‐PURB (5 µg), anti‐AGO2 (5 µg) or IgG (5 µg) antibodies at 4 °C for 2 h. After being washed with 1 × Buffer A twice, the beads were incubated with cell lysates at 4 °C overnight and then washed with 1 × Buffer B through vortex for 2 min 5 times. Next, the immunoprecipitated RNAs were extracted, reverse‐transcribed, and detected by qRT–PCR.

### Co‐Immunoprecipitation (co‐IP) Assay

Cells were treated with MG132 (MCE, USA) and lysed by NP‐40 Lysis Buffer (Beyotime, China). The cell lysates were incubated with anti‐p53 antibodies at 4 °C overnight, and then captured by protein A+G magnetic beads (Beyotime, China) at room temperature for 2 h the next day. The immunoprecipitated proteins were separated by SDS‐PAGE and detected by western blot.

### Immunohistochemical (IHC)

IHC was performed routinely. Briefly, tumor tissue sections were deparaffinized and rehydrated, followed by antigen retrieval by citrate buffer at 95 °C and blocked by Immunol Staining Blocking Buffer (Beyotime, China). The sections were incubated with primary antibodies at 4 °C overnight, and then incubated with the secondary antibody at 37 °C for 30 min. DAB Horseradish Peroxidase Color Development Kit (Beyotime, China) was used for the chromogenic reaction.

### Immunofluorescence

Immunofluorescence was performed following the FISH assay and cells were blocked with 5% BSA. Subsequently, cells were incubated with primary antibodies at 4 °C overnight and then fluorescent secondary antibodies at room temperature for 2 h. Nuclei were stained with DAPI. The samples were photographed by THUNDER Imaging Systems (Leica, Germany).

### Cleavage under targets and release using nuclease (CUT&RUN)

CUT&RUN was performed using a Hyperactive pG‐MNase CUT&RUN Assay Kit for Illumina (Vazyme, China) according to the manufacturer's instructions. In brief, RBE cells were prepared and followed with the incubation of Concanavalin A–coated beads, anti‐PURB antibody, and pG‐MNase Enzyme in order. Then CaCl2 was added to activate the enzyme for fragmentation reaction. The reaction was stopped by Stop Buffer and DNA fragments were extracted. After that, the library for sequencing was constructed using the DNA fragments products followed by the procedures of DNA damage repair & end preparation, adapter ligation, and library amplification. The products were purified by VAHTS DNA Clean Beads (Vazyme, China) and sequenced on an Illumina novaseq 150PE.

### Chromatin Isolation by RNA Purification (ChIRP)

A ChIRP Kit (BersinBio, China) was used for ChIRP assays according to the manufacturer's instructions. Briefly, 1 × 10^7^ RBE cells were prepared and cross‐linked by 1% formaldehyde. Then cells were lysed and chromatins were sonicated into 100 ∼ 500 bp fragments with the length being confirmed by agarose gel electrophoresis. Subsequently, the samples were incubated with a biotinylated circUGP2 probe in a Hybridization Buffer and then bound to the Streptavidin Beads. After being washed with wash buffer, the purified DNA was isolated using the phenol‐chloroform method and detected by PCR.

### Dual‐luciferase Reporter Assay

For PURB and ADGRB1 promoter binding site verification, wild‐type (WT) and mutant (MT) ADGRB1 promoter fragments were constructed and inserted into the upstream of luciferase reporter gene in the reporter plasmid pRL‐SV40 (GenePharma, China). HEK‐293T cells were co‐transfected with WT or MT plasmid, combined with PURB overexpression plasmid or control vector. To confirm the interaction between miR‐3191‐5p and circUGP2 or ADGRB1 3′‐UTR, WT and MT circUGP2 and ADGRB1 3′‐UTR fragments were constructed and inserted into the downstream of luciferase reporter gene. HEK‐293T cells were co‐transfected with WT or MT plasmid, combined with miR‐3191‐5p mimics or control. After 48 h, a Dual‐Luciferase Reporter System Kit (Vazyme, China) was used to examine firefly luciferase activities, which were normalized against Renilla luciferase activities.

### Animal Experiment

The animal experimental protocol was approved by the Committee on the Use of Live Animals in Teaching and Research, Nanjing Medical University. 4‐week‐old male BALB/c nude mice were purchased from Charles River and bred in the Animal Core Facility of Nanjing Medical University, under a 12 h light/dark cycle with free access to water and food. For the establishment of a subcutaneous tumor xenograft model, 5 × 10^6^ stable QBC939 or RBE cells were suspended in 100 µL phosphate‐buffered saline (PBS) and injected into the groin of mice. The mice were euthanized 4 weeks later and then tumors were collected and weighed. The volumes of tumors were calculated with the formula (width^2^ × length)/2. To construct a liver metastasis model, mice were anesthetized and 5 × 10^6^ stable QBC939 or RBE cells suspended in 100 µL PBS were injected into the spleen parenchyma. The mice were sacrificed after 4 weeks and liver samples were collected. For the treatment with LNP systems, LNP‐NC or LNP‐circUGP2 (plasmids: 3 mg k^−1^g) was injected through the tail vein twice weekly for 4 weeks. After being euthanized, the mice tumor, major organs, and blood samples were immediately collected for further analysis.

### Preparation and Characterization of Lipid Nanoparticles (LNPs)

The LNPs were synthesized as previously described.^[^ ^35^ ^]^ DOTAP (RuixiBiotech, China), DPPC (MCE, USA), DSPE‐PEG2000‐folate (RuixiBiotech, China), and cholesterol (MCE, USA) in a molar ratio of 50:10:38.5:1.5 were dissolved in a chloroform/methanol mixture (3:1). The mixture was then dried by a rotary evaporator under vacuum condition at 42 °C with a speed of 100 r/min until a uniform thin lipid film was formed. Plasmids were dissolved in sodium acetate solution and added into the lipid film with a 1:10 molar ratio of the plasmids to the lipid materials. Subsequently, by adding several glass beads, the lipid film was shaken and hydrated until it completely detached from the bottle wall. The obtained LNPs were extruded through a 200 nm polycarbonate membrane and concentrated by a 100‐kDa ultrafiltration centrifuge tube. Dynamic light scattering (DLS) and polydispersity index (PdI) of LNPs were measured by NS‐90Z Plus (OMEC, China). The transmission electron microscopy (TEM) images of LNPs were taken by JEM 1200EX (JEOL, Japan).

### Biodistribution of LNPs

In order to investigate the biodistribution of LNPs, DiD dye (MCE, USA) was added to the lipid mixture at the first step to label LNPs. Subcutaneous tumor‐bearing mice were injected with DiD‐labeled LNPs through the tail vein and imaged by an in vivo fluorescence imaging system (PerkinElmer, USA) at the indicated times.

### Statistical Analysis

Data were present as mean ±SD and analyzed with GraphPad Prism 8.0 and SPSS 21.0 software. Continuous variables were analyzed by paired or unpaired Student's t‐test. A chi‐square test was applied for categorical variables. Survival data were evaluated by the Kaplan‐Meier method and compared by the log‐rank test. Pearson's correlation test was used to examine the correlation between ADGRB1 and circUGP2 or miR‐3191‐5p. Sample size (n) for each statistical analysis was mentioned in the Figure legends. A value of *p <0.05, **p < 0.01, or ***p <0.001 were considered statistically significant.

### Ethics Approval and Consent to Participate

This study was performed in accordance with the Declaration of Helsinki and was approved by the Ethics Committee of the First Affiliated Hospital of Nanjing Medical University (2019‐SR‐133). Each patient had signed the informed consent ahead of participation in this study. The animal experimental protocol was approved by the Committee on the Use of Live Animals in Teaching and Research, Nanjing Medical University (IACUC‐2311064).

## Conflict of Interest

The authors declare no conflict of interest.

## Author Contributions

R.X.C., S.C.L., X.C.K., and Y.R.W. contributed equally as joint first authors Rui Xiang Chen, Shuo Chen Liu, and Yi Rui Wang performed most of the experiments, analyzed the data, and wrote the manuscript. Xue Chun Kan constructed the LNP system and performed the relative experiments. Tian Lin Wang, Ji Fei Wang, and Kuang Heng Shi provided support for animal models and IHC staining. Chang Li, Shi Long Fan, and Xiao Xu provided support for cellular function experiments. Yan An Lan Chen and Tao Zhou provided support for CUT&RUN assays. Wang Jie Jiang, Jiang Chang, Yao Dong Zhang, Ming Yu Wu, and Yue Yu provided helpful advice. Chang Xian Li and Xiang Cheng Li designed the present study, reviewed the manuscript, and provided grant support. All authors have read and approved the final version of the manuscript.

## Supporting information

Supporting Information

## Data Availability

The data that support the findings of this study are available from the corresponding author upon reasonable request.,

## References

[1] D. Moris , M. Palta , C. Kim , P. J. Allen , M. A. Morse , M. E. Lidsky , CA Cancer J Clin 2023, 73, 198. 36260350 10.3322/caac.21759

[2] J. W. Valle , R. K. Kelley , B. Nervi , D. Y. Oh , A. X. Zhu , Lancet 2021, 397, 428. 33516341 10.1016/S0140-6736(21)00153-7

[3] V. Mazzaferro , A. Gorgen , S. Roayaie , M. Droz Dit Busset , G. Sapisochin , J Hepatol 2020, 72, 364. 31954498 10.1016/j.jhep.2019.11.020

[4] T. F. Greten , R. Schwabe , N. Bardeesy , L. Ma , L. Goyal , R. K. Kelley , X. W. Wang , Nat. Rev. Gastroenterol. Hepatol. 2023, 20, 349. 36697706 10.1038/s41575-022-00741-4 PMC12468729

[5] H. Wang , M. Guo , H. Wei , Y. Chen , Signal Transduct Target Ther 2023, 8, 92. 36859359 10.1038/s41392-023-01347-1 PMC9977964

[6] M. R. O'Dell , J. L. Huang , C. L. Whitney‐Miller , V. Deshpande , P. Rothberg , V. Grose , R. M. Rossi , A. X. Zhu , H. Land , N. Bardeesy , A. F. Hezel , Cancer Res. 2012, 72, 1557. 22266220 10.1158/0008-5472.CAN-11-3596 PMC3306549

[7] H. Zhang , D. Zhu , Z. Zhang , S. Kaluz , B. Yu , N. S. Devi , J. J. Olson , E. G. Van Meir , Oncogene 2020, 39, 1041. 31582835 10.1038/s41388-019-1036-7 PMC7780546

[8] D. Zhu , S. Osuka , Z. Zhang , Z. R. Reichert , L. Yang , Y. Kanemura , Y. Jiang , S. You , H. Zhang , N. S. Devi , D. Bhattacharya , S. Takano , G. Y. Gillespie , T. Macdonald , C. Tan , R. Nishikawa , W. G. Nelson , J. J. Olson , E. G. Van Meir , Cancer Cell 2018, 33, 1004. 29894688 10.1016/j.ccell.2018.05.006 PMC6002773

[9] L. S. Kristensen , M. S. Andersen , L. V. W. Stagsted , K. K. Ebbesen , T. B. Hansen , J. Kjems , Nat. Rev. Genet. 2019, 20, 675. 31395983 10.1038/s41576-019-0158-7

[10] L. L. Chen , Nat. Rev. Mol. Cell Biol. 2020, 21, 475. 32366901 10.1038/s41580-020-0243-y

[11] C. X. Liu , L. L. Chen , Cell 2022, 185, 2016. 35584701 10.1016/j.cell.2022.04.021

[12] J. Li , D. Sun , W. Pu , J. Wang , Y. Peng , Trends Cancer 2020, 6, 319. 32209446 10.1016/j.trecan.2020.01.012

[13] J. Du , T. Lan , H. Liao , X. Feng , X. Chen , W. Liao , G. Hou , L. Xu , Q. Feng , K. Xie , M. Liao , X. Chen , J. Huang , K. Yuan , Y. Zeng , Mol Cancer 2022, 21, 18. 35039066 10.1186/s12943-021-01482-9 PMC8762882

[14] Q. Chen , H. Wang , Z. Li , F. Li , L. Liang , Y. Zou , H. Shen , J. Li , Y. Xia , Z. Cheng , T. Yang , K. Wang , F. Shen , J Hepatol 2022, 76, 135. 34509526 10.1016/j.jhep.2021.08.027

[15] D. Witzigmann , J. A. Kulkarni , J. Leung , S. Chen , P. R. Cullis , R. van der Meel , Adv Drug Deliv Rev 2020, 159, 344. 32622021 10.1016/j.addr.2020.06.026 PMC7329694

[16] P. R. Cullis , M. J. Hope , Mol. Ther. 2017, 25, 1467. 28412170 10.1016/j.ymthe.2017.03.013 PMC5498813

[17] H. Li , Z. Q. Zhou , Z. R. Yang , D. N. Tong , J. Guan , B. J. Shi , J. Nie , X. T. Ding , B. Li , G. W. Zhou , Z. Y. Zhang , Hepatology 2017, 66, 136. 28194813 10.1002/hep.29116

[18] C. E. Wu , C. Y. Huang , C. P. Chen , Y. R. Pan , J. W. Chang , J. S. Chen , C. N. Yeh , J. Lunec , Cancers (Basel) 2021, 13, 3876. 34359777 10.3390/cancers13153876 PMC8345393

[19] J. Wang , T. Zhao , J. Chen , L. Kang , Y. Wei , Y. Wu , L. Han , L. Shen , C. Long , S. Wu , G. Wei , J. Hazard. Mater. 2021, 406, 124316. 33162236 10.1016/j.jhazmat.2020.124316

[20] M. J. Duffy , N. C. Synnott , S. O'Grady , J. Crown , Semin. Cancer Biol. 2022, 79, 58. 32741700 10.1016/j.semcancer.2020.07.005

[21] Z. Tang , C. Li , B. Kang , G. Gao , C. Li , Z. Zhang , Nucleic Acids Res. 2017, 45, W98. 28407145 10.1093/nar/gkx247 PMC5570223

[22] L. A. Ferris , R. J. Kelm , J. Cell. Biochem. 2019, 120, 5835. 30387171 10.1002/jcb.27869 PMC6382573

[23] C. M. Livi , P. Klus , R. Delli Ponti , G. G. Tartaglia , Bioinformatics 2016, 32, 773. 26520853 10.1093/bioinformatics/btv629 PMC4795616

[24] M. Zuker , Nucleic Acids Res. 2003, 31, 3406. 12824337 10.1093/nar/gkg595 PMC169194

[25] Y. Zhang , J. Wang , Y. Xiao , J. Mol. Biol. 2022, 434, 167452. 35662453 10.1016/j.jmb.2022.167452

[26] Y. Yan , H. Tao , J. He , S. Y. Huang , Nat. Protoc. 2020, 15, 1829. 32269383 10.1038/s41596-020-0312-x

[27] P. R. Pandey , J. H. Yang , D. Tsitsipatis , A. C. Panda , J. H. Noh , K. M. Kim , R. Munk , T. Nicholson , D. Hanniford , D. Argibay , X. Yang , J. L. Martindale , M. W. Chang , S. W. Jones , E. Hernando , P. Sen , S. De , K. Abdelmohsen , M. Gorospe , Nucleic Acids Res. 2020, 48, 3789. 31980816 10.1093/nar/gkaa035 PMC7144931

[28] K. C. Chang , S. D. Diermeier , A. T. Yu , L. D. Brine , S. Russo , S. Bhatia , H. Alsudani , K. Kostroff , T. Bhuiya , E. Brogi , D. J. Pappin , C. F. Bennett , F. Rigo , D. L. Spector , Nat. Commun. 2020, 11, 6438. 33353933 10.1038/s41467-020-20207-y PMC7755919

[29] X. Messeguer , R. Escudero , D. Farré , O. Núñez , J. Martínez , M. M. Albà , Bioinformatics 2002, 18, 333. 11847087 10.1093/bioinformatics/18.2.333

[30] S. E. McGeary , K. S. Lin , C. Y. Shi , T. M. Pham , N. Bisaria , G. M. Kelley , D. P. Bartel , Science 2019, 366, eaav1741. 31806698 10.1126/science.aav1741 PMC7051167

[31] V. Agarwal , G. W. Bell , J. W. Nam , D. P. Bartel , Elife 2015, 4, e05005. 26267216 10.7554/eLife.05005 PMC4532895

[32] M. Rehmsmeier , P. Steffen , M. Hochsmann , R. Giegerich , RNA 2004, 10, 1507. 15383676 10.1261/rna.5248604 PMC1370637

[33] C. Sticht , C. De La Torre , A. Parveen , N. Gretz , PLoS One 2018, 13, e0206239. 30335862 10.1371/journal.pone.0206239 PMC6193719

[34] Y. Lu , P. S. Low , Adv Drug Deliv Rev 2002, 54, 675. 12204598 10.1016/s0169-409x(02)00042-x

[35] Z. W. Chen , F. P. Kang , C. K. Xie , C. Y. Liao , G. Li , Y. D. Wu , H. Y. Lin , S. C. Zhu , J. F. Hu , C. F. Lin , Y. Huang , Y. F. Tian , L. Huang , Z. W. Wang , S. Chen , Adv. Sci. (Weinh) 2023, 10, e2303814. 37789644 10.1002/advs.202303814 PMC10646249

[36] Y. Gu , Y. Wang , L. He , J. Zhang , X. Zhu , N. Liu , J. Wang , T. Lu , L. He , Y. Tian , Z. Fan , Mol Cancer 2021, 20, 132. 34649567 10.1186/s12943-021-01435-2 PMC8515748

[37] Y. Xu , S. Zhang , X. Liao , M. Li , S. Chen , X. Li , X. Wu , M. Yang , M. Tang , Y. Hu , Z. Li , R. Yu , M. Huang , L. Song , J. Li , Mol Cancer 2021, 20, 98. 34325714 10.1186/s12943-021-01394-8 PMC8320207

[38] H. J. Kim , K. Yang , K. Kim , Y. J. Lee , S. Lee , S. Y. Ahn , Y. H. Ahn , J. L. Kang , Cell Mol. Immunol. 2022, 19, 1373. 36241874 10.1038/s41423-022-00930-w PMC9708692

[39] J. Cao , X. Liu , Y. Yang , B. Wei , Q. Li , G. Mao , Y. He , Y. Li , L. Zheng , Q. Zhang , J. Li , L. Wang , C. Qi , Angiogenesis 2020, 23, 325. 32020421 10.1007/s10456-020-09707-z

[40] D. Zhu , S. B. Hunter , P. M. Vertino , E. G. Van Meir , Cancer Res. 2011, 71, 5859. 21724586 10.1158/0008-5472.CAN-11-1157 PMC3165103

[41] S. Yuan , X. Wang , S. Hou , T. Guo , Y. Lan , S. Yang , F. Zhao , J. Gao , Y. Wang , Y. Chu , J. Shi , T. Cheng , W. Yuan , Leukemia 2022, 36, 370. 34465864 10.1038/s41375-021-01392-1 PMC8807395

[42] S. M. Cork , B. Kaur , N. S. Devi , L. Cooper , J. H. Saltz , E. M. Sandberg , S. Kaluz , E. G. Van Meir , Oncogene 2012, 31, 5144. 22330140 10.1038/onc.2012.1 PMC3355202

[43] S. A. Khan , H. C. Thomas , M. B. Toledano , I. J. Cox , S. D. Taylor‐Robinson , Liver Int 2005, 25, 704. 15998419 10.1111/j.1478-3231.2005.01106.x

[44] H. Zhu , H. Gao , Y. Ji , Q. Zhou , Z. Du , L. Tian , Y. Jiang , K. Yao , Z. Zhou , J. Hematol. Oncol. 2022, 15, 91. 35831864 10.1186/s13045-022-01314-3 PMC9277894

[45] S. Singh , T. Sinha , A. C. Panda , Wiley Interdiscip Rev RNA 2023, 3876, e1820. 10.1002/wrna.1820 37783567

[46] Y. Xu , K. Leng , Y. Yao , P. Kang , G. Liao , Y. Han , G. Shi , D. Ji , P. Huang , W. Zheng , Z. Li , J. Li , L. Huang , L. Yu , Y. Zhou , X. Jiang , H. Wang , C. Li , Z. Su , S. Tai , X. Zhong , Z. Wang , Y. Cui , Hepatology 2021, 73, 1419. 32750152 10.1002/hep.31493

[47] J. E. Zhou , L. Sun , Y. Jia , Z. Wang , T. Luo , J. Tan , X. Fang , H. Zhu , J. Wang , L. Yu , Z. Yan , J. Control. Rel. 2022, 350, 298. 10.1016/j.jconrel.2022.08.033 36002054

