# Supplementary material for: CircUGP2 Suppresses Intrahepatic Cholangiocarcinoma Progression via p53 Signaling Through Interacting With PURB to Regulate ADGRB1 Transcription and Sponging miR‐3191‐5p
Source: Adv Sci (Weinh). 2024 Aug 9;11(38):2402329. doi: 10.1002/advs.202402329 (PMC11481218; doi:10.1002/advs.202402329)
Supplement: Supplementary file 1 — Supporting Information [file ADVS-11-2402329-s001.pdf]

ADVANCED SCIENCE
Open Access

Supporting Information

for *Adv. Sci.*, DOI 10.1002/advs.202402329

CircUGP2 Suppresses Intrahepatic Cholangiocarcinoma Progression via p53 Signaling Through Interacting With PURB to Regulate ADGRB1 Transcription and Sponging miR-3191-5p

Rui Xiang Chen, Shuo Chen Liu, Xue Chun Kan, Yi Rui Wang, Ji Fei Wang, Tian Lin Wang, Chang Li, Wang Jie Jiang, Yan An Lan Chen, Tao Zhou, Shi Long Fan, Jiang Chang, Xiao Xu, Kuang Heng Shi, Yao Dong Zhang, Ming Yu Wu, Yue Yu, Chang Xian Li and Xiang Cheng Li**

CircUGP2 Suppresses Intrahepatic Cholangiocarcinoma Progression via p53 Signaling through Interacting with PURB to Regulate ADGRB1 Transcription and Sponging miR-3191-5p

*Rui Xiang Chen [1, #], Shuo Chen Liu[1, #], Xue Chun Kan[2, #], Yi Rui Wang[1, #], Ji Fei Wang[1], Tian Lin Wang[1], Chang Li[1], Wang Jie Jiang[1], Yan An Lan Chen[1], Tao Zhou[1], Shi Long Fan[1], Jiang Chang[1], Xiao Xu[1], Kuang Heng Shi[1], Yao Dong Zhang[1], Ming Yu Wu[3], Yue Yu[1], Chang Xian Li[1, *], Xiang Cheng Li[1, 3, *].*

1 Hepatobiliary Center, The First Affiliated Hospital of Nanjing Medical University; Key Laboratory of Liver Transplantation, Chinese Academy of Medical Sciences; NHC Key Laboratory of Living Donor Liver Transplantation (Nanjing Medical University), Nanjing, Jiangsu Province, China.

2 School of Medicine, Southeast University, Nanjing, Jiangsu Province, China.

3 The Affiliated Wuxi People's Hospital of Nanjing Medical University, Wuxi People's Hospital, Wuxi Medical Center, Nanjing Medical University, Wuxi, Jiangsu Province, China.

#These authors contributed equally as joint first authors

***Corresponding author:**

Prof. Xiang Cheng Li

Hepatobiliary Center, First Affiliated Hospital of Nanjing Medical University, 300 Guangzhou Road, Nanjing, Jiangsu Province, China. E-mail: drxcli@njmu.edu.cn

Dr. Chang Xian Li

Hepatobiliary Center, First Affiliated Hospital of Nanjing Medical University, 300 Guangzhou Road, Nanjing, Jiangsu Province, China. E-mail: drlicx@njmu.edu.cn

Table S1. Primers, siRNAs and probes used in this study.

Name	Sense (5’-3’)
circUGP2-Forward	GGATGGATTTCGGAAGCTATTTC
circUGP2-Reverse	GAGACATTGCTTTGCTAAGATCGA
UGP2-Forward	ATGTCTCAAGATGGTGCTTCTCA
UGP2-Reverse	GGTGTGCTCAAATTCATGTGATG
GAPDH-Forward	GAACGGGAAGCTCACTGG
GAPDH-Reverse	GCCTGCTTCACCACCTTCT
U6-Forward	CTCGCTTCGGCAGCACA
U6-Reverse	AACGCTTCACGAATTTGCGT
GAPDH (divergent)-Forward	TGTACCATCAATAAAGTACCCTGTG
GAPDH (divergent)-Reverse	AAATCCGTTGACTCCGACCT
MDM2-Forward	GAATCATCGGACTCAGGTACATC
MDM2-Reverse	TCTGTCTCACTAATTGCTCTCCT
CDKN1A-Forward	TGTCCGTCAGAACCCATGC
CDKN1A-Reverse	AAAGTCGAAGTTCCATCGCTC
GADD45A-Forward	GAGAGCAGAAGACCGAAAGGA
GADD45A-Reverse	CAGTGATCGTGCGCTGACT
SFN-Forward	TGACGACAAGAAGCGCATCAT
SFN-Reverse	GTAGTGGAAGACGGAAAAGTTCA
Maspin-Forward	AATTCGGCTTTTGCCGTTGAT
Maspin-Reverse	TGTCACCTTTAGCACCCACTT
BBC3-Forward	GACCTCAACGCACAGTACGAG
BBC3-Reverse	AGGAGTCCCATGATGAGATTGT
BAX-Forward	CCCGAGAGGTCTTTTTCCGAG
BAX-Reverse	CCAGCCCATGATGGTTCTGAT
PURB-Forward	GCCATCACCGTACCCTTCAAA
PURB-Reverse	CCCTCTGTCGTTCCTGGATTT
ADGRB1-Forward	GCGGCGCTACACTCTCTAC
ADGRB1-Reverse	GCACCTCGTCGAAGCTCTC
TP53-Forward	CAGCACATGACGGAGGTTGT
TP53-Reverse	TCATCCAAATACTCCACACGC
FOLR1-Forward	GCTCAGCGGATGACAACACA
FOLR1-Reverse	CCTGGCCCATGCAATCCTT
GAPDH (mouse)-Forward	AGGTCGGTGTGAACGGATTTG
GAPDH (mouse)-Reverse	TGTAGACCATGTAGTTGAGGTCA
FOLR1 (mouse)-Forward	TGATGTGGATGGCCGAATGTG
FOLR1 (mouse)-Reverse	GTCGTGTAAATTGTCCTCAGGG
ADGRB1 promoter 0~500-Forward	GCGCTCGCGGGGATTT
ADGRB1 promoter 0~500-Reverse	GACTCGATCCGGCGAGTTG
ADGRB1 promoter -400~0-Forward	GCGCGTGTGTGCATTTTATC
ADGRB1 promoter -400~0-Reverse	GGGAGCCAGAGACTGCATTA
ADGRB1 promoter -800~-400-Forward	CCCACTCAGCCCGCTCT

ADGRB1 promoter -800~-400-Reverse	CAGCACCCAGTGGAGGTCT
ADGRB1 promoter -1200~-800-Forward	AAGTCAAAGGAGGGCGGATG
ADGRB1 promoter -1200~-800-Reverse	GAGATGTTGAGGGGAGTGGC
ADGRB1 promoter -1600~-1200-Forward	CGTGACCTGCTGAGATGCTT
ADGRB1 promoter -1600~-1200-Reverse	GATGCTTCTGCCTCAACCCT
ADGRB1 promoter -2000~-1600-Forward	CAAGAGGTTGGACCGGGAAG
ADGRB1 promoter -2000~-1600-Reverse	CCTCAAGGCTGATCTGTGGC
ADGRB1 promoter -5400~-5000-Forward	GAACTGGGATGGCTGGAGAG
ADGRB1 promoter -5400~-5000-Reverse	AGAGACAAGGCACAACCTGG
hsa-miR-326-RT	GTCGTATCCAGTGCAGGGTCCGAGG TATTCGCACTGGATACGACCTGGAG
hsa-miR-326-Forward	CGCCTCTGGGCCCTTC
hsa-miR-3191-5p-RT	GTCGTATCCAGTGCAGGGTCCGAGG TATTCGCACTGGATACGACTGGAAG
hsa-miR-3191-5p-Forward	GCGCTCTCTGGCCGTCTAC
hsa-miR-6732-5p-RT	GTCGTATCCAGTGCAGGGTCCGAGG TATTCGCACTGGATACGACGGCCAG
hsa-miR-6732-5p-Forward	GCGTAGGGGGTGGCAGG
hsa-miR-6798-5p-RT	GTCGTATCCAGTGCAGGGTCCGAGG TATTCGCACTGGATACGACCCCAAG
hsa-miR-6798-5p-Forward	CAGGGGGATGGGCGAG
miRNA-Reverse	AGTGCAGGGTCCGAGGTATT
si circUGP2-1	CCUGAAGAUUCGAUCUUAGTT
si circUGP2-2	UGAAGAUUCGAUCUUAGCATT
sh circUGP2	TGAAGATTCGATCTTAGCA
si PURB	GCGAGAACCGCAAGUACUATT
si ADGRB1	GGAUCCUGGUGUUCAACAATT
hsa-miR-3191-5p mimic	CUCUCUGGCCGUCUACCUUCCA
hsa-miR-3191-5p inhibitor	UGGAAGGUAGACGGCCAGAG
Biotin labeled circUGP2 probe	GCTAAGATCGAATCTTCAGG-/3bio/
Biotin labeled NC probe	AGTGATGACCGACTATAGTC-/3bio/

Table S2. Antibodies used in this study.

Antibodies Name	Company
Anti-p53	Abcam (ab17990)
Anti-Ki-67	Abcam (ab16667)
Anti-GAPDH	Abcam (ab9485)
Anti-AKT	Cell Signaling (9272)
Anti-p-AKT	Cell Signaling (9271)
Anti-AGO2	Cell Signaling (2897)
Anti-Flag	Cell Signaling (14793)
Anti-p21	Proteintech (10355-1-AP)
Anti-PURB	Proteintech (18128-1-AP)
Anti-MDM2	Proteintech (27883-1-AP)
Anti-ubiquitin	Proteintech (10201-2-AP)
Anti-F4/80	Proteintech (28463-1-AP)
Anti-ADGRB1	Absin (abs147973)

Figure S1. CircUGP2 inhibits ICC progression in vitro and in vivo. (A) CCK-8 assays performed in QBC939 cells and RBE cells transfected with circUGP2 siRNAs or plasmid. (n=3) (B) EdU assays performed in circUGP2 knockdown or overexpression cells. (scale bar, 50μm) (n=3) (C) Transwell assays were preformed to evaluate the migration and invasion in QBC939 and RBE cells. (original magnification, 100X) (n=3) (D) Wound healing assays preformed in these cell lines. (original magnification, 40X) (n=3) (E) Comparisons of tumor weights and volumes between circUGP2 overexpression or knockdown group and the corresponding control groups. (n=5) (F) Representative images of H&E and Ki-67 staining of the subcutaneous xenograft tumors. (scale bar, 50μm) (G) Comparisons of liver metastasis count between circUGP2 overexpression or knockdown group and the corresponding control groups. (n=4-5) (H) H&E staining of metastatic nodules in mice liver sections. (scale bar, 200μm) Data were present as mean ±SD. *$P<0.05$, **$P<0.01$, ***$P<0.001$. Unpaired Student's t test (A-E, G).

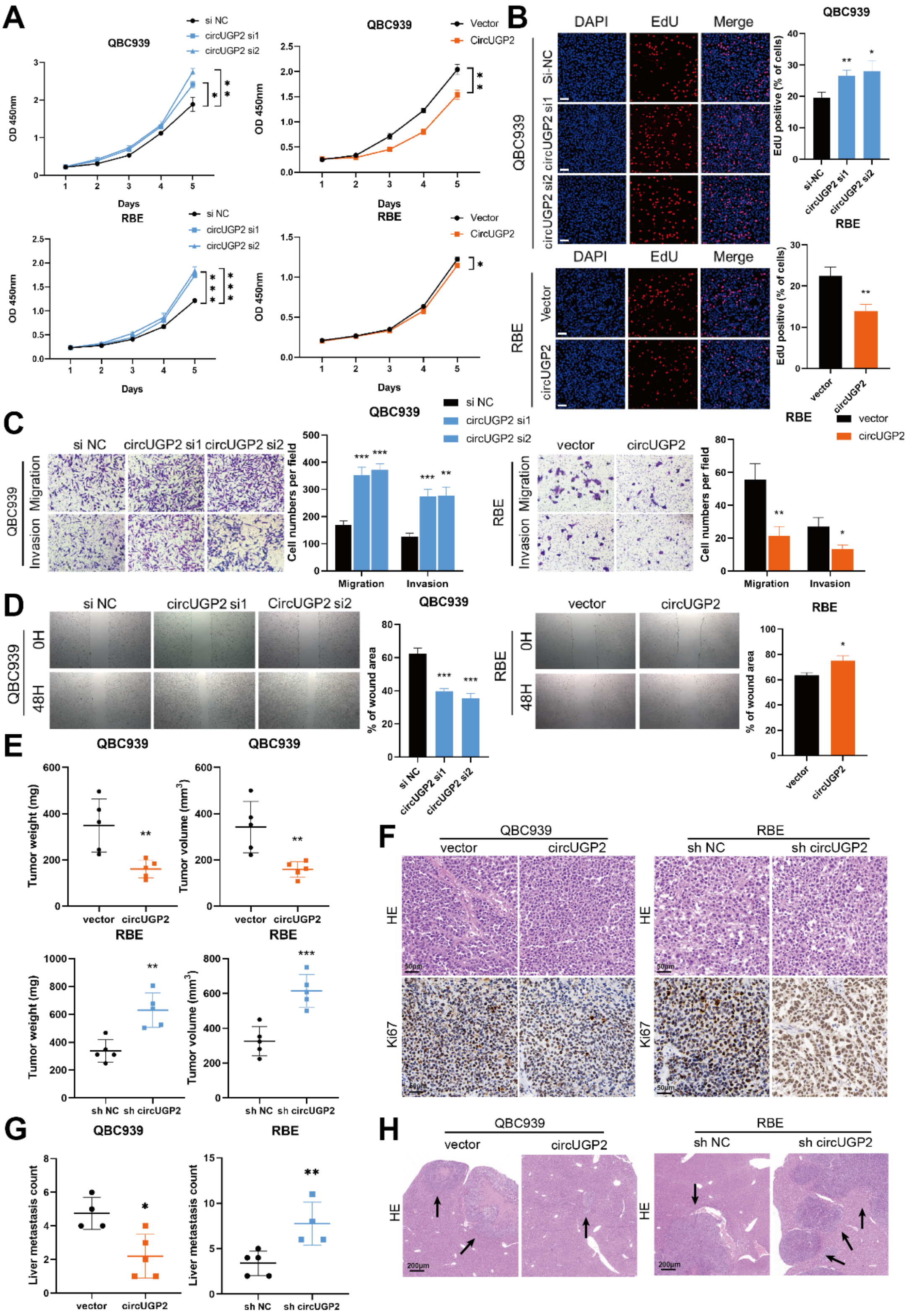
A
QBC939
si NC
circUGP2 si1
circUGP2 si2
OD 450nm
Days
Vector
CircUGP2
RBE
B
DAPI
EdU
Merge
Si-NC
circUGP2 si1
circUGP2 si2
EdU positive (% of cells)
si-NC
Vector
circUGP2
vector
C
Migration
Invasion
Cell numbers per field
D
0H
48H
CircUGP2 si2
% of wound area
E
Tumor weight (mg)
Tumor volume (mm3)
sh NC
sh circUGP2
F
HE
Ki67
G
Liver metastasis count
H

Figure S2. CircUGP2 activates the p53 signaling pathway in ICC. (A) Heatmap of differentially expressed genes in RBE cells with circUGP2 overexpression by RNA-seq. (B) KEGG pathway analysis of differentially expressed genes in RBE cells with circUGP2 overexpression and control group. (C) GSEA analysis of PI3K-Akt signaling pathway. (D) Western blot analysis showed the levels of AKT and p-AKT in QBC939 and RBE cells. (E) Clone formation assays performed in QBC939 cells with the indicated the treatment. (n=3) (F) Transwell assays performed in QBC939 cells. (original magnification, 100X) (n=3) Data were present as mean ±SD. *P<0.05, **P<0.01, ***P<0.001. Unpaired Student’s t test (E-F).

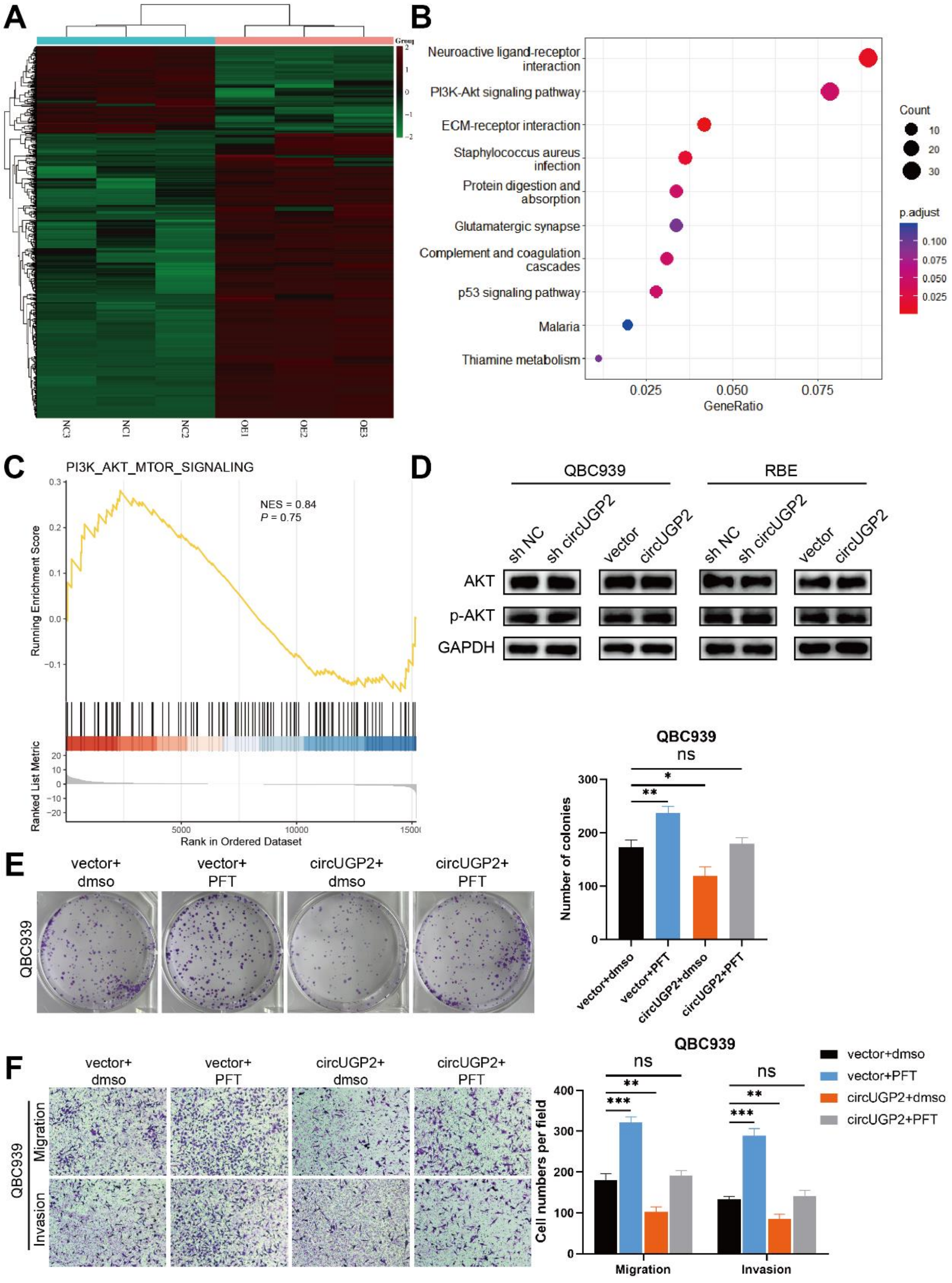

Figure S3. CircUGP2 interacts with PURB to regulate ADGRB1 transcription. (A) Clone formation assays performed in RBE cells with the indicated the treatment. (n=3) (B) Transwell assays performed in RBE cells. (original magnification, 100X) (n=3) (C) Diagram of the 3D structure of PURB (left) and circUGP2 (middle), and the molecular docking between them (right). (D) The mRNA levels of ADGRB1 were examined by qRT–PCR in PURB overexpression and knockdown cells. (n=3) (E) Dual-luciferase report assays performed in HEK-293T with the indicated treatment. (n=3) Data were present as mean ±SD. *P<0.05, **P<0.01, ***P<0.001. Unpaired Student's t test (A-B, D-E).

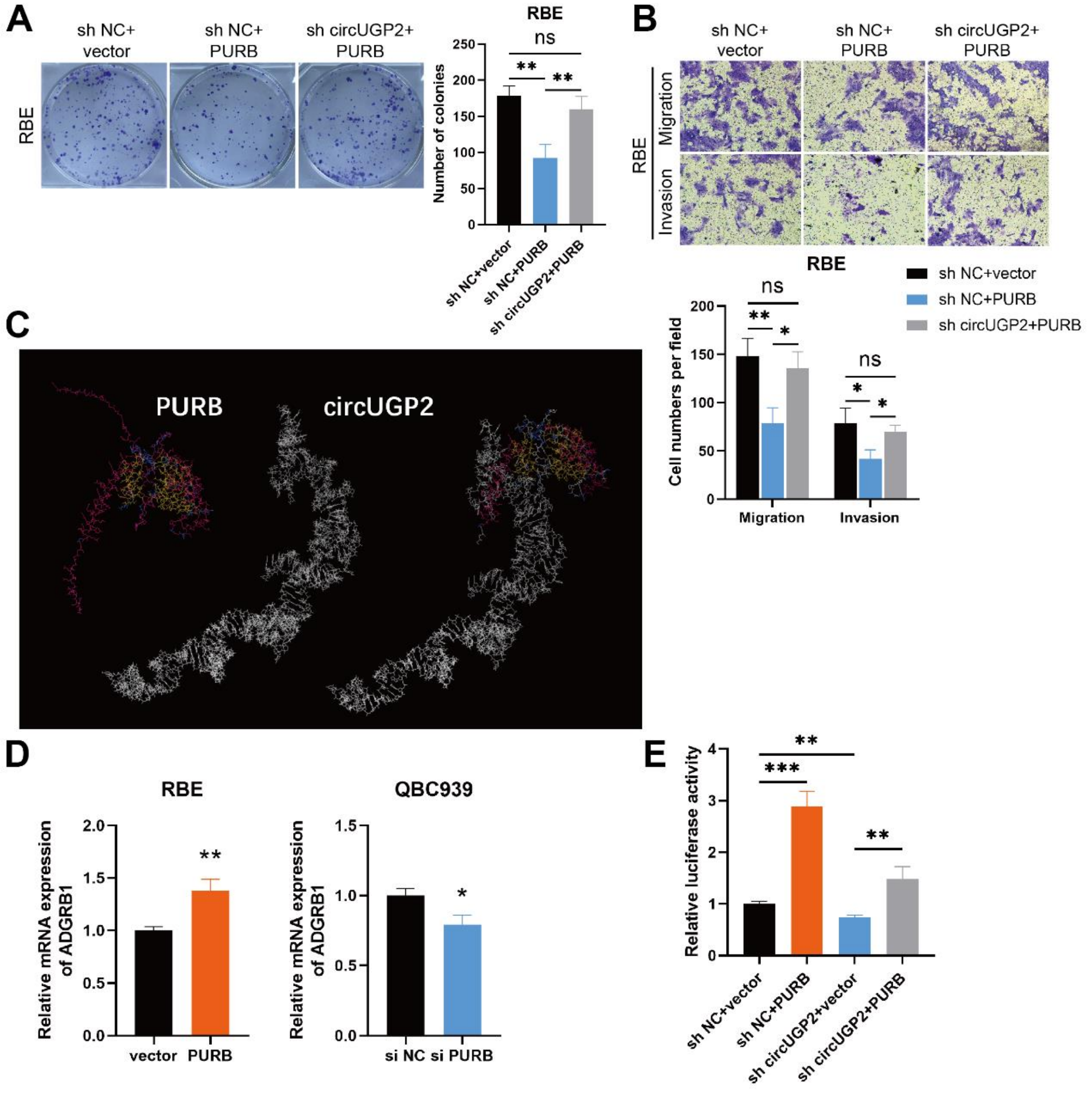

Figure S4. CircUGP2 inhibited ICC progression through sponging miR-3191-5p. (A) CCK-8 assays performed in QBC939 cells and RBE cells with the indicated treatment. (n=3) (B) Clone formation assays performed in QBC939 cells with the indicated treatment. (n=3) (C) Transwell assays performed in QBC939 cells. (original magnification, 100X) (n=3) (D) Wound healing assays preformed in QBC939 cells. (original magnification, 40X) (n=3) (E) Dual-luciferase report assays performed in HEK-293T with the indicated treatment. (n=3) Data were present as mean ±SD. *P<0.05, **P<0.01, ***P<0.001. Unpaired Student's t test (A-E).

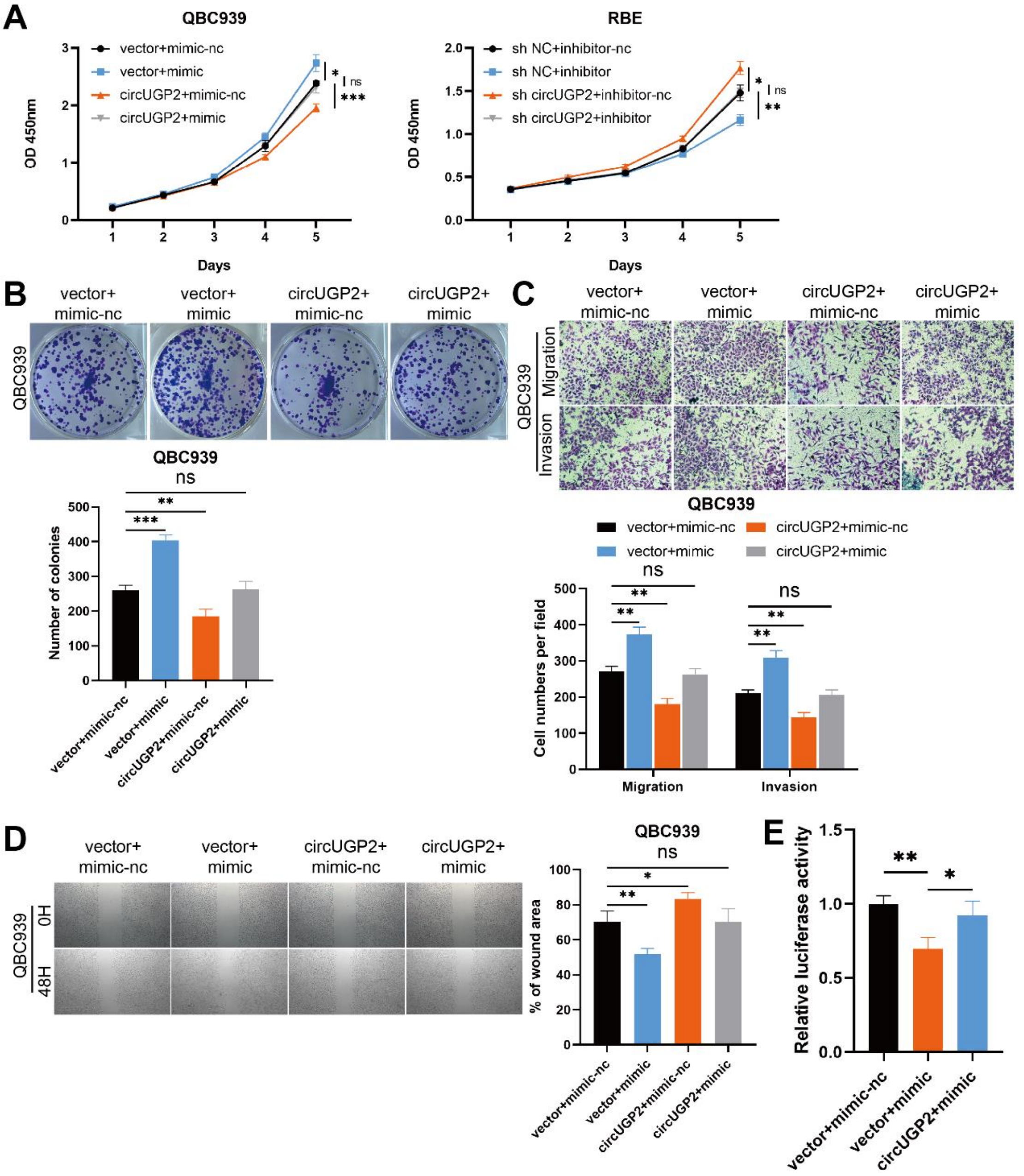

Figure S5. CircUGP2 activates ADGRB1/p53 axis to suppress ICC progression. (A) Co-IP assays were performed to determine the p53 polyubiquitination levels in QBC939 cells with MDM2 and circUGP2 overexpression. (B) The mRNA levels of TP53 were examined by qRT–PCR in ADGRB1 overexpression and knockdown cells. (n=3) (C) Western blot analysis showed the levels of p53 in QBC939 cells after CHX treatment for the indicated times. (D) Clone formation assays performed in QBC939 cells with the indicated treatment. (n=3) (E) Wound healing assays preformed in QBC939 cells. (original magnification, 40X) (n=3) (F) Transwell assays performed in QBC939 cells. (original magnification, 100X) (n=3) Data were present as mean ±SD. *P<0.05, **P<0.01, ***P<0.001. Unpaired Student's t test (B, D-F).

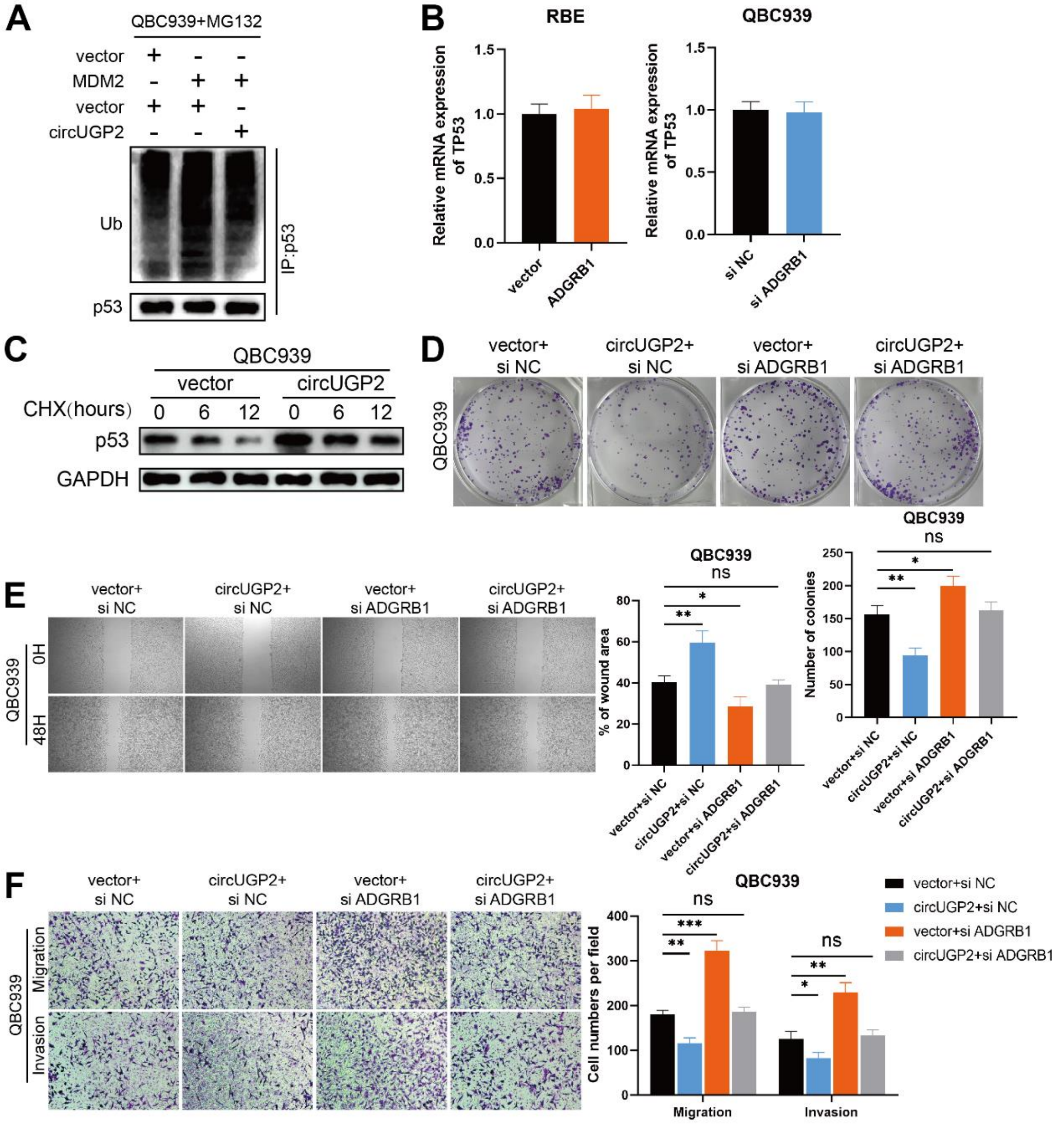

Figure S6. Therapeutic strategy using LNP-encapsulated circUGP2 plasmid reveals anti-tumor effects. (A) The mRNA levels of FOLR1 (FR alpha) in HiBEC cells, RBE cells, mice primary hepatocytes and Kupffer cells were examined by qRT–PCR. (n=3) (B) Fluorescence images showed the biodistribution of DiD-labeled LNP in tumors and major organs. (C) The levels of circUGP2 in the subcutaneous tumors after LNP treatment. (n=3) (D) Western blot analysis showed the levels of p53 and p21 in the subcutaneous tumors after LNP treatment. (E) qRT-PCR analysis of p53 target gene expressions in the subcutaneous tumors after LNP treatment. (n=3) (F) Comparisons of liver metastasis count between groups with the indicated treatment. (n=4-5) (G) The body weights of subcutaneous tumor-bearing mice after the indicated treatments. (n=5) (H) H&E staining of the major organs of the subcutaneous tumor-bearing mice. (scale bar, 50μm) (I) Serum ALT, AST and CREA levels of the subcutaneous tumor-bearing mice following the indicated treatment. (n=5) Data were present as mean ±SD. *P<0.05, **P<0.01, ***P<0.001. Unpaired Student's t test (A, C, E-G, I).

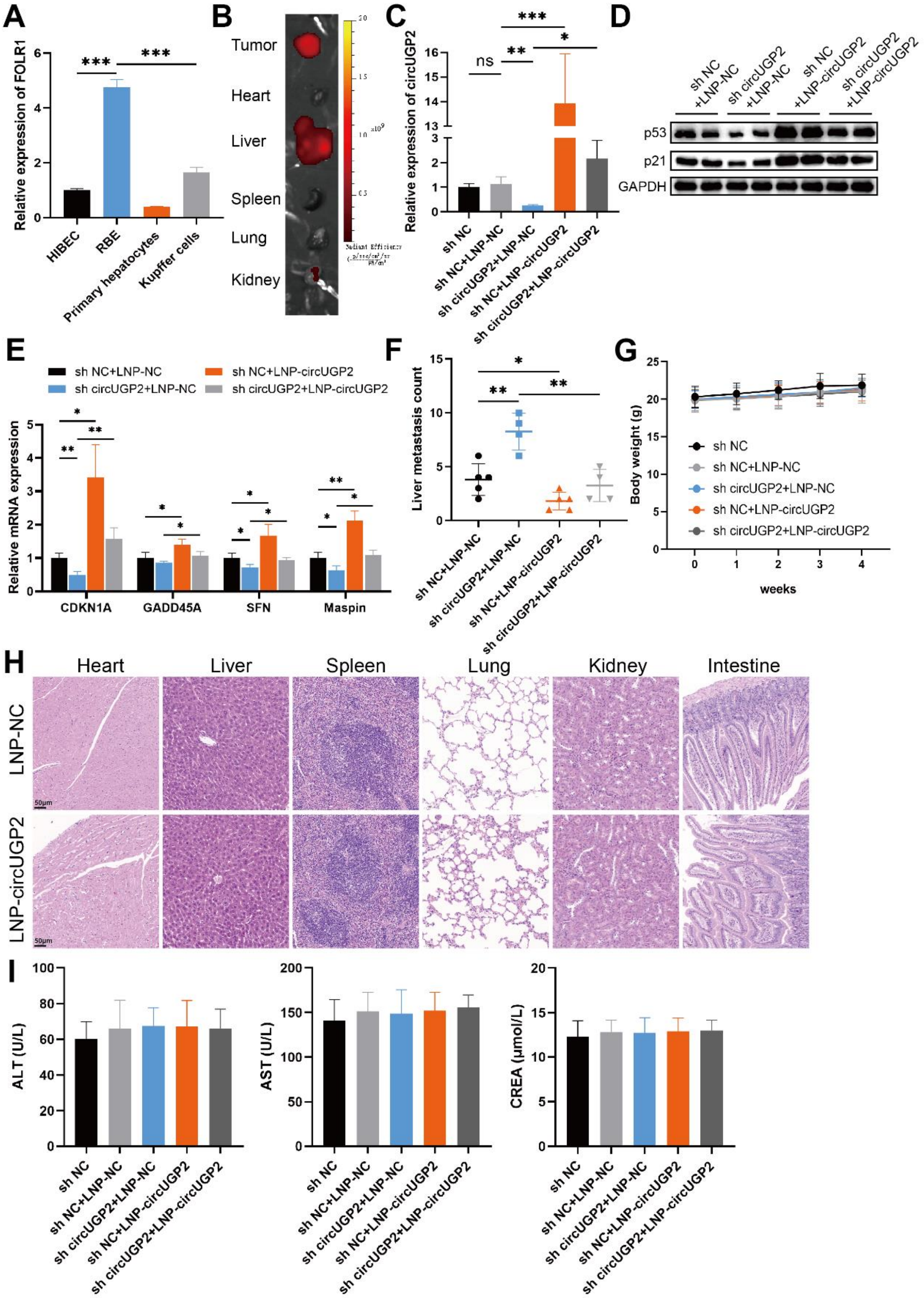
A
Relative expression of FOLR1
6
4
2
0
***
***
HIBEC
RBE
Primary hepatocytes
Kupffer cells
B
Tumor
Heart
Liver
Spleen
Lung
Kidney
C
Relative expression of circUGP2
16
15
14
13
3
2
1
0
ns
**
***
*
sh NC
sh NC+LNP-NC
sh circUGP2+LNP-NC
sh NC+LNP-circUGP2
sh circUGP2+LNP-circUGP2
D
sh NC +LNP-NC
sh circUGP2 +LNP-NC
sh NC +LNP-circUGP2
sh circUGP2 +LNP-circUGP2
p53
p21
GAPDH
E
sh NC+LNP-NC
sh circUGP2+LNP-NC
sh NC+LNP-circUGP2
sh circUGP2+LNP-circUGP2
Relative mRNA expression
5
4
3
2
1
0
CDKN1A
GADD45A
SFN
Maspin
F
Liver metastasis count
15
10
5
0
*
**
**
sh NC+LNP-NC
sh circUGP2+LNP-NC
sh NC+LNP-circUGP2
sh circUGP2+LNP-circUGP2
G
Body weight (g)
25
20
15
10
5
0
sh NC
sh NC+LNP-NC
sh circUGP2+LNP-NC
sh NC+LNP-circUGP2
sh circUGP2+LNP-circUGP2
0
1
2
3
4
weeks
H
Heart
Liver
Spleen
Lung
Kidney
Intestine
LNP-NC
LNP-circUGP2
50μm
I
ALT (U/L)
100
80
60
40
20
0
AST (U/L)
200
150
100
50
0
CREA (μmol/L)
20
15
10
5
0
sh NC
sh NC+LNP-NC
sh circUGP2+LNP-NC
sh NC+LNP-circUGP2
sh circUGP2+LNP-circUGP2
